# Shape-Controlled Iron–Paraffin Composites as γ- and X-ray Shielding Materials Formable by Warmth-of-Hands-Derived Plasticity

**DOI:** 10.1021/acsaenm.3c00557

**Published:** 2023-12-01

**Authors:** Jolanta Sobczak, Adrian Truszkiewicz, Emil Korczeniewski, Aleksandra Cyganiuk, Artur P. Terzyk, Anna Kolanowska, Rafał G. Jędrysiak, Sławomir Boncel, Gaweł Żyła

**Affiliations:** † Doctoral School of the Rzeszów University of Technology, Rzeszów University of Technology, 35-959 Rzeszów, Poland; ‡ Department of Photomedicine and Physical Chemistry, Medical College of University of Rzeszow, University of Rzeszow, Warzywna 1A Street, 35-310 Rzeszów, Poland; § Faculty of Chemistry, Physicochemistry of Carbon Materials Research Group, Nicolaus Copernicus University in Torun, Gagarin Street 7, 87-100 Torun, Poland; ∥ Department of Organic Chemistry, Bioorganic Chemistry and Biotechnology, Silesian University of Technology, 44-100 Gliwice, Poland; ⊥ Biotechnology Centre, Silesian University of Technology, 44-100 Gliwice, Poland; # Centre for Organic and Nanohybrid Electronics, Silesian University of Technology, 44-100 Gliwice, Poland; ∇ Department of Physics and Medical Engineering, Rzeszow University of Technology, 35-959 Rzeszow, Poland

**Keywords:** iron, paraffin, nanocomposites, microcomposites, γ-rays, X-rays, shielding

## Abstract

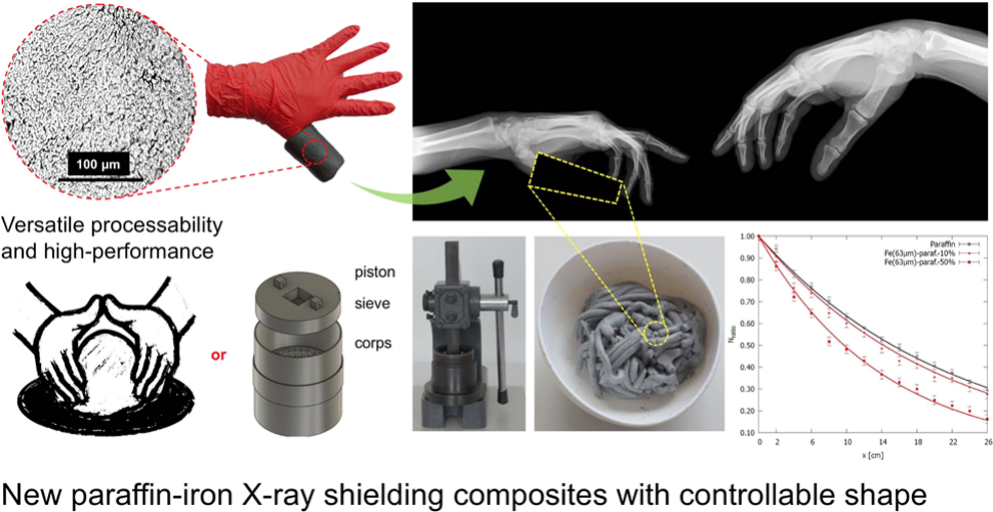

The design of shielding materials against ionizing radiation while simultaneously displaying enhanced multifunctional characteristics remains challenging. Here, for the first time, we present moldable paraffin-based iron nano- and microcomposites attenuating γ- and X-radiation. The moldability was gained by the warmth-of-hands-driven plasticity, which allowed for obtaining a specific shape of the composites at room temperature. The manufactured composites contained iron particles of various sizes, ranging from 22 nm to 63 μm. The target materials were widely characterized using XRD, NMR, Raman, TGA, SEM, and EDX. In the case of microcomposites, the shielding properties were developed at two concentrations: 10 and 50 wt %. The statistically significant results indicate that the iron particle size has a negligible effect on the shielding properties of the nano- and microcomposites. On the other hand, the higher iron particle contents significantly affected the attenuating ability, which emerged even as superior to the elemental aluminum in the X-ray range: at a 70 kV anode voltage, the half value layer was 6.689, 1.882, and 0.462 cm for aluminum, paraffin + 10 wt % Fe 3.5–6.5 μm, and paraffin + 50 wt % Fe 3.5–6.5 μm microcomposites, respectively. Importantly, the elaborated methodology—in situ cross-verified in the hospital studies recording real-life sampling—opens the pathway to high-performance, eco-friendly, lightweight, and recyclable shields manufactured via fully reproducible and scalable protocols.

## Introduction

The design of a constructive shield against ionizing radiation is one of the main requirements in contact with radioactive isotopes, starting with the medical sector (radiology, diagnostic imaging), through nuclear reactors, and ending with research and development centers and radiation physics laboratories.^1,2^ The selection of a proper shield for a given radioactive source is conditioned by the type of radiation itself, i.e., emitted energy, so that basic parameters such as thickness, linear/mass attenuation coefficient, effective removal cross sections, and half/tenth value length of the selected shield have met the requirements of radiation protection. Narrowing down the subject to γ and X-ray radiation, the adopted traditional solutions offer shields made of metal elements with a high atomic number, such as lead, iron, or bismuth. The manufactured shielding materials, despite their effectiveness in radiation attenuation, are not free from disadvantages, which include toxicity, heavyweight, cracks, impracticality, difficult recyclability, and inability to be easily accessible or produced in the desired geometry. With this in mind, several attempts were made to improve those conventional solutions so that apart from suppressing the dose to the maximum permissible value, the manufactured shields would have other desirable physicochemical properties; therefore, nano- and microcomposites were introduced.

The established term of composite is understood as a material created with at least two components evenly distributed throughout the volume of the matrix, whose physical properties are the result of the individual components leading to the synergetic functionalities.^3^ The concept of a nanocomposite is not only in line with the adopted definition but also has uniquely broadened this definition to contain various systems made of different components (organic, inorganic, amorphous, etc.) mixed with nanometer scale additions. In broadly understood nanotechnology dedicated to radiation protection, it can be observed that the implementation of nano- and microsized heavy elements (bismuth, tungsten, and lead) as the main ingredients is becoming increasingly prevalent in research conducted by physicists, chemists, and materials engineers.

It is undeniable that iron is the most ubiquitous of the transition metals, and considering its mass, it is the most common chemical element on Earth. It comes in the form of a shiny, silvery metal that undergoes passivation reactions in organic solvents: concentrated nitric acid (HNO_3_)^4^ and concentrated sulfuric acid (H_2_SO_4_).^5^ Due to its physicochemical properties, iron is found in a group of the most commonly used metals, among others, in the construction, aviation, energy, and fuel industries. It is also used to protect against ionizing radiation. An iron thick shield was studied in the last century for application as a reactor shield, as it is presented in Ref. (6), where a 70 cm thick iron shield was examined against neutron- and γ-rays with the reactor YAYOI in Japan. Moreover, the literature shows that there are studies where iron with various additions was also included. Using advanced casting and thermomechanical treatments, iron was investigated as a matrix phase, with two types of yttrium oxide (Y_2_O_3_) nanopowders added for high-temperature nuclear reactor applications,^7^ and a subject of research was high-chromium iron-based alloys, as an alternative to austenitic stainless steels, for the advanced nuclear reactor application as well.^8^

According to the concept of Richard Feynman (‘There’s Plenty of Room at the Bottom’, lecture presented at the American Physical Society at Caltech, in 1959), research on the properties of iron nanoparticles is continuously performed; however, considering nano- and microcomposites, the presence of this element is incipient. Sathish et al.^9^ proposed a nanocomposite synthesized via combustion from a mixture of Fe(NO_3_)_2_·9H_2_O, Ba(NO_3_)_2_, and Ni(NO_3_)_2_·6H_2_O as oxidants and/or metallic precursors and urea (CH_4_N_2_O) as a fuel. The radiation shielding properties (X-rays and γ-rays) were established experimentally using different sources: ^137^Cs (0.6615 MeV), ^60^Co (1.173 and 1.332 MeV), ^22^Na (0.511, 0.081 MeV), and ^133^Ba (0.276 and 0.356 MeV) and theoretically with WinXCom software (from 1 keV to 100 GeV energy range) for various thicknesses: 5, 10, 15, 20, and 25 mm. The linear attenuation coefficients obtained experimentally varied for individual energy values and ranged from 2.16 ± 0.11 for ^56^Ba (0.081 MeV) to 0.25 ± 0.01 for ^60^Co (1.332 MeV); therefore, as the energy value increased, the shielding quality of nanocomposite samples decreased. Moreover, the outcomes showed that above 356 keV, both experimental and calculated values agreed. In another work, El-Khatib et al.^10^ manufactured a mortar made from cement, marble, and nano- and microsized iron slag (wastes from the marble and steel industry) to investigate the shielding characteristics depending on the iron particle size. The samples, with dimensions of 30 mm in diameter and 5 mm in height, were experimentally examined using ^241^Am, ^133^Ba, ^137^Cs, ^60^Co, and ^152^Eu point sources, and the obtained values were compared to the calculated values from the XCOM program. It was stated that both micro- and nanosized iron slag improved attenuating properties, while nanosized iron slag displayed superior shielding performance. Similarly, Al-Rajhi et. al^11^ explored the shielding properties of an iron slag nanopowder (ISNP). The experimental and calculated values of the mass attenuation coefficients were in good agreement with each other and confirmed a decrease in the attenuation coefficient with higher photon energy. Nevertheless, the authors emphasized that their value is relatively large compared to those of some traditional concrete shields. In another work,^12^ high-density polyethylene (HDPE)-based composite, doped with different ratios of iron oxide (α-Fe_2_O_3_) and aluminum metal (Al) nanoparticles, was proposed as a γ-ray shielding material. The prepared composites contained 60% HDPE and 40% α-Fe_2_O_3_, 60% HDPE with 30% α-Fe_2_O_3_ and 10% Al, and 60% HDPE with 20% α-Fe_2_O_3_ and 20% Al. The attenuation properties were theoretically obtained using the WinXCom and MCNP5 programs, as well as experimentally using ^131^Cs and ^60^Co radioactive sources. The presence of these additives improved the shielding properties of the HDPE composites; however, it was emphasized that the fillers caused significant attenuating properties at photon energies lower than 0.1 MeV; at 0.662 MeV, the mass attenuation coefficients were μ/ρ (cm^2^ g^–1^) 0.111, 0.110, and 0.099 cm^2^ g^–1^, and at 1.33 MeV, they were 0.077, 0.076, and 0.075 cm^2^ g^–1^ accordingly for 60% HDPE and 40% α-Fe_2_O_3_, 60% HDPE with 30% α-Fe_2_O_3_ and 10% Al, and 60% HDPE with 20% α-Fe_2_O_3_ and 20% Al composites.

Another promising radiation shielding material was a poly(vinyl alcohol) (PVA) film doped with magnetite Fe_3_O_4_ nanoparticles.^13^ The samples obtained by the coprecipitation method were examined using ^60^Co, ^137^Cs, and ^22^Na sources. Again, the obtained linear attenuation coefficient of the PVA nanocomposite was compared to the linear attenuation coefficient of lead, and it was found that at 0.662 MeV photon energy, the nanocomposite has a shielding ability of nearly 59% that of lead.

From the perspective of moldability, an interesting material that is easily processable toward protection against γ and X-radiation is paraffin. Its use is known for its protection against neutron radiation,^14^ while it can also be extended to ionizing radiation. Exploring it in more detail, paraffin is a mixture of solid alkanes (saturated organic compounds) with the general formula C*_n_*H_2*n*+2_ (*n* > 10) obtained from the crude oil distillation process. It occurs as a white to yellowish, odorless, tasteless, and waxy solid, whose physical properties are affected by the length of the hydrocarbon chain and molecular weight distribution.^15^ Its use is found in pharmaceutical and cosmetics factories, the paper industry, household chemicals, the production of candles and grave lights, the manufacturing of gloss pastes, the thickening of lubricating oils, and as a thermally and electrically insulating material.^16^ The solution with partial paraffin implementation in γ shielding materials is present in phase change core/shell microcapsules.^17,18^ On the other hand, the idea of a new composite material should also include the aspect of recycling, which not only could lead to the production of a new product but, above all, would reduce overexploited natural deposits/raw materials and generated garbage. This aspect was notably marked during the 1972 Stockholm Conference of the United Nations under the slogan ‘We only have one earth’, where the concept of environmental protection policy was emphasized^19^ as a result of the development of civilization and, above all, growing consumerism. Nowadays, this topic is still relevant; therefore, incessant improvement in waste management procedures is vital. It is confirmed in scientific articles, for instance, recycling materials into nanocomposites with graphene addition was lately summarized in a review paper,^20^ nanocomposites containing recycled components, and dedicated to different applications were presented,^21−23^ as examples. Narrowing down to nano- and microcomposites directed to γ and X-ray protection, and the authors also use recycled ingredients;^24−26^ however, the possible step of separating the ingredients for their reuse is not encountered in experimental papers.

There is a wide body of studies in the area of X- and γ-radiation shielding properties of various component-based composites. For instance, nonmutagenic and nonteratogenic bismuth and tungsten,^27^ as elements with higher shielding ability than that of iron, are potential candidates for active fillers in the X-ray and γ-radiation shielding composites. However, considering the values of their atomic masses, iron has a lower density. Moreover, our bodies contain natural reservoirs of iron, such as blood or spleen, with the latter storing iron in the form of ferritin or bilirubin; hence, the toxicity of iron is low. To the best of our knowledge, there are no data about iron–paraffin nano- and microcomposites, where paraffin would play a role of a chemically inert matrix. Here, we report comprehensive studies on the manufacturing and radiation protection efficiency of accessible and easily scalable composite materials. Importantly, those materials are moldable due to the plasticity achievable by the warmth of hands. Their high anti-X- and γ-radiation performance, proved by their high versatility accompanied by the overall characteristics, was cross-verified as a function of the size of iron nano- and microparticles, forming a 3D network as the filler dispersed in paraffin (of the well-tuned characteristics) as the continuous phase. Eventually, the Fe–paraffin microcomposite emerged as the most prospective and economic composite in terms of manufacturing (iron as the large-scale and abundant metal in the form of microparticles in place of more expensive nanoparticles) and recycling (with practically endless options of reforming and reshaping coupled with an easy process to recover ingredients).

## Materials and Methods

### Preparation and Characterization of Paraffin-Based Iron Nano- and Microcomposites

Iron nano- and microparticles were purchased from Nanografi (Ankara, Turkey) and Selkat (Kraków, Poland), respectively. The following sizes were declared by the manufacturers: 22 nm, 30–40 nm, 60–70 nm, 90–100 nm, 3.5–6.5 μm, and 63 μm. All of the particles were used to prepare the shielding composites. It is well-known that the shielding properties of iron are not as efficient as those of other high-density metals (i.e., lead and bismuth); however, iron is less toxic, so the scope of possible applications of iron-based materials is much wider. Commercially available (Orlen Południe, Trzebinia, Poland) paraffin was used, and its physical and chemical characteristics (declared by the manufacturer) are collected in Table 1.

**Table 1:** Physicochemical Properties of the Paraffin Used in This Study

appearance	white, odorless solid
melting point/freezing point	max 348.15 K
boiling point	min 573.15 K
flash point	min 453.15
temperature of self-ignition	min 523.15 K
pH	∼7 (aqueous solution)
kinematic viscosity	6–10 × 10^–6^ m^2^ s^–1^ at 100 °C (373.15 K)
density	∼755 kg m^–3^ at 100 °C (373.15 K)

**Figure 1:**
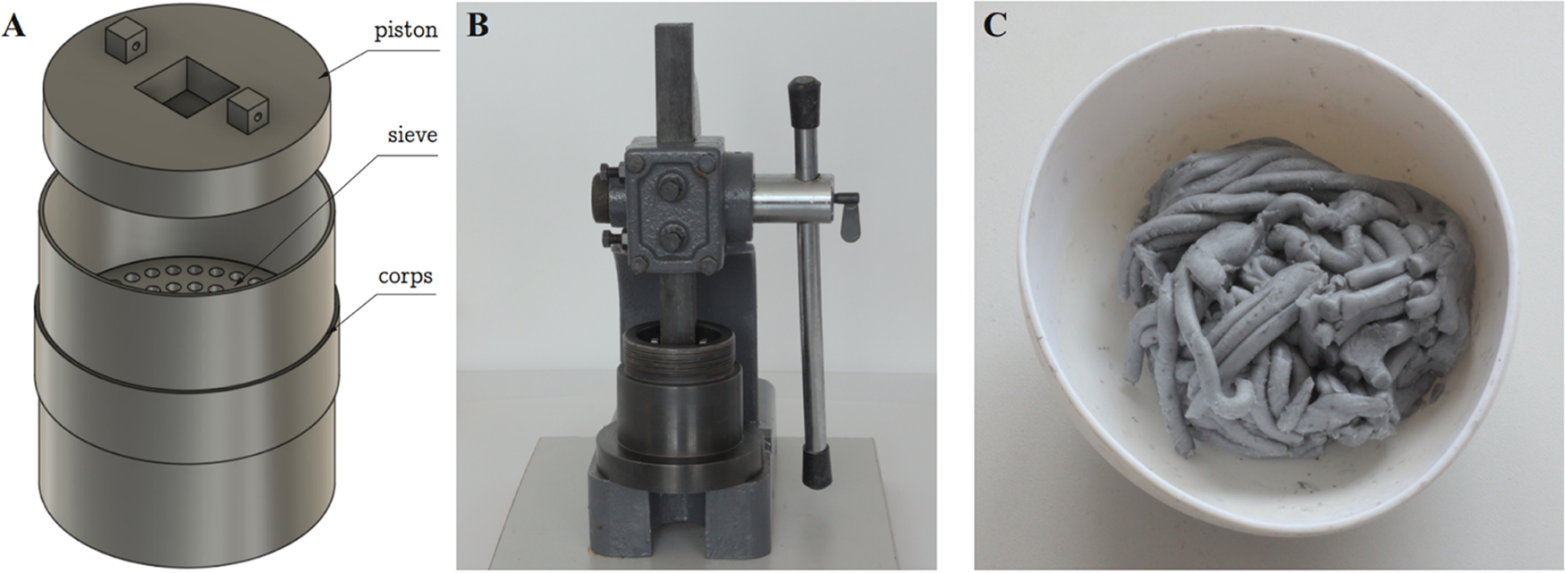
Pictures of a manual press: a design (A), the mixing system (B), and the final mixed microcomposite (C), exemplified by the 10 wt % Fe 63 μm microcomposite.

The nanocomposite samples were manufactured using iron powder (with the average particle sizes given above) with a 10 wt % concentration. The microcomposites (particle sizes: 3.5, 6.5, and 63 μm) were developed with two various concentrations: 10 and 50 wt %. For this purpose, the appropriate amount of iron micro- or nanoparticles and paraffin was weighed. The weighing was performed using an analytical balance WTC 2000 (Radwag, Radom, Poland) with an accuracy of 0.01 g. Second, the sample was placed in a Goldbrunn 450 vacuum dryer (Berlin, Germany) at an ambient temperature higher than the melting point of paraffin (120 °C), in order to eliminate air bubbles from the paraffin. At the next stage, after the paraffin has been crystallized, the sample is subjected to the previously designed cold mixing on a hand press for about 50 min. Figure 1 presents the projected cold mixing system (Figure 1A), the manufactured design with a hand press (Figure 1B), and the final blended microcomposite (Figure 1C).

**Figure 2:**
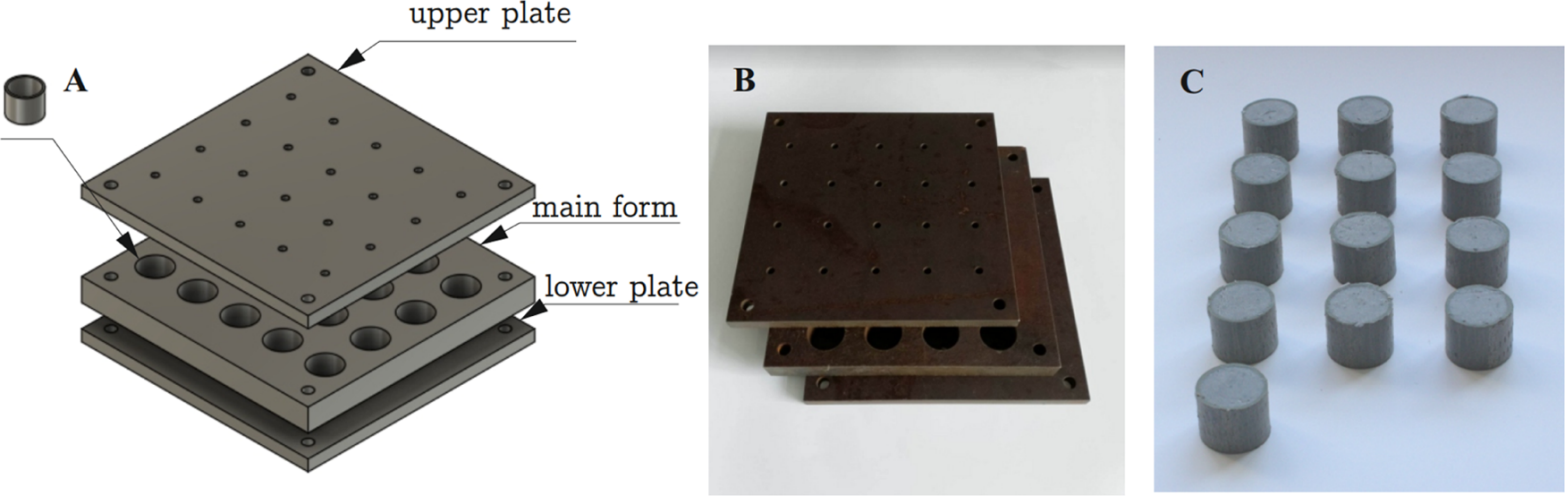
Arrangement of three molds to produce filled rings with micro- and nanocomposites: isometric projection (A), photograph of manufactured form and plates (B), and photograph of the microcomposite samples (C).

Then, the samples were placed with a 30% weight allowance in 2 cm high rings, and these, in turn, were placed in metal form limited at the top and at the bottom by another plate. Afterward, a set of three molds was subjected to pressure on a hand press, and any excess material from the individual rings was removed through holes (5 mm in diameter) in the upper and lower plates. The designed main form, with a lower and upper plate, is depicted in Figure 2A, while the manufactured form and plates are shown in Figure 2B. Based on such a solution for the experimental research, thirteen (13) rings filled with iron micro- and nanocomposites were produced (Figure 2C). Importantly, no plasticizing agents were used during sample preparation. The picture summarizing the sequence of the individual stages of microcomposite production is shown in Figure S1 in the Supporting Information, while their ability to change shape at room temperature is shown in the Movie, also attached in the Supporting Information.

X-ray diffraction analysis (XRD) spectra were acquired using a Philips X’Pert instrument in transmission mode over a 5–100° 2θ range. An X’Celerator Scientific detector with a Cu anode (Malvern, U.K.) was used to assign the scanning speed as 2.1° min^–1^. The diffraction profiles were corrected to consider a background, followed by smoothing cycles. The Raschinger method was used to remove the contribution of Kα2.

Proton nuclear magnetic resonance (^1^H NMR) and carbon-13 nuclear magnetic resonance (^13^C NMR) spectra were recorded using a Varian Inova 600 MHz instrument. δ-Values are presented in parts per million (ppm) in relation to tetramethylsilane (TMS) as the internal standard compound.

Raman spectra (aperture 50 μm × 1000 μm) were measured by using a SENTERRA micro-Raman system (Bruker, Germany). The spectra (at least three for each sample) were obtained using a laser (532 nm) in the range of 60–1500 cm^–1^. The power of the laser was fine-tuned from the list of 2, 5, and 20 mW; the laser exposure times were tuned from the list of 40, 50, and 60 s of double shots. The 300/200/100 one s shots were also used for the lowest power value. Si(100) was used as a substrate for the grains. For paraffin and paraffin-iron composites, one- or five s double shots with a power of 20 mW on Au substrate were used.

Thermogravimetric analysis (TGA) curves were recorded under both nitrogen and air atmospheres at a heating rate of 20 °C min^–1^ and a final temperature of 800 °C by applying TGA 8000 (PerkinElmer).

Scanning electron microscopy (SEM) and energy-dispersive X-ray spectroscopy (SEM/EDX) data were acquired using three models: Quanta 3D FEG (FEI Company, Hillsboro, Oregon) equipped with a BSE detector, 1430 VP (LEO Electron Microscopy Ltd., Cambridge, U.K.) equipped with an energy-dispersive X-ray spectrometer (EDS) Quantax 200 conjugated with an XFlash 4010 detector (Bruker AXS GmbH, Karlsruhe, Germany), and Phenom Pro desktop SEM (Phenom-World Holding B.V., Eindhoven, Netherlands) (the last one for the paraffin and its composites). To estimate the real sizes of nano- and microparticles, a series of SEM images and ImageJ (imagej.net/software/imagej/) image processing program were used.

### γ-Shielding Measurements

**Figure 3:**
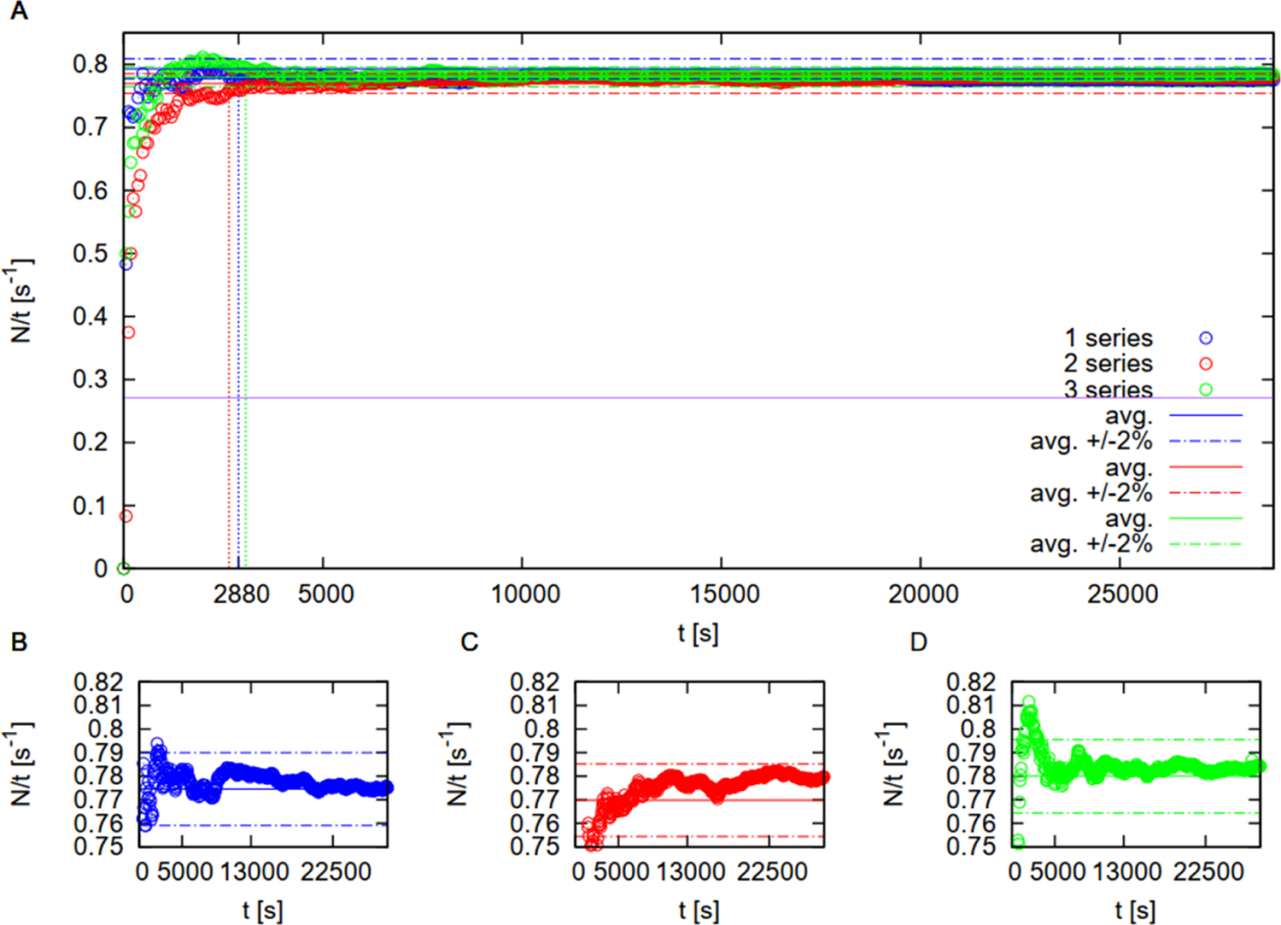
Dependence of the activity, *N*/*t* (s^–1^), of the ^60^Co source on time *t* (s) at the measuring point. The solid lines show the average values obtained with each series, while the upper and lower dashed horizontal lines present the deviation ±2% of the result. Dashed vertical lines indicate the time after which each measuring point lies within the 2% deviation of the average. The horizontal purple line presents the background activity. Measurement data from the three-measurement series (A), and single measurement series from the individual series (B–D).

In order to experimentally obtain shielding properties of paraffin-based micro- and nanocomposites, a point source ^60^Co (activity: 427.5 ± 8.2 kBq, energy of emitted photons: 1.173 and 1332 MeV, half-life 5.2711 ± 0.0008 years) was chosen, and as a detector, the Geiger–Müller (G-M) counter (3B Scientific Physics, Hamburg, Germany), with a dead time of 90 μs, was employed. Prior to the measurements, the characteristics of the used γ radiation source were verified to determine the time of measurement that could be considered reliable. For this purpose, three series of ^60^Co source activity measurements were performed. In each series, the γ photons detected in the G-M counter were counted at a time of 8 h within a 1 min step. After that time, the average photon number at the time (activity) was calculated, as presented in Figure 3. The average number of counts collected within the G-M counter during the whole 8 h period was calculated, and it was checked to see if the deviation between the so-obtained results and the average values was lower than 2%. It was consequently concluded that a plateau in the number of counts was achieved after 2880 s (48 min). In order to ensure the stability of the acquisition of the count number for further measurements, the time of 48 min was extended by 25%, so that in the studies, the acquisition time was 1 h.

**Figure 4:**
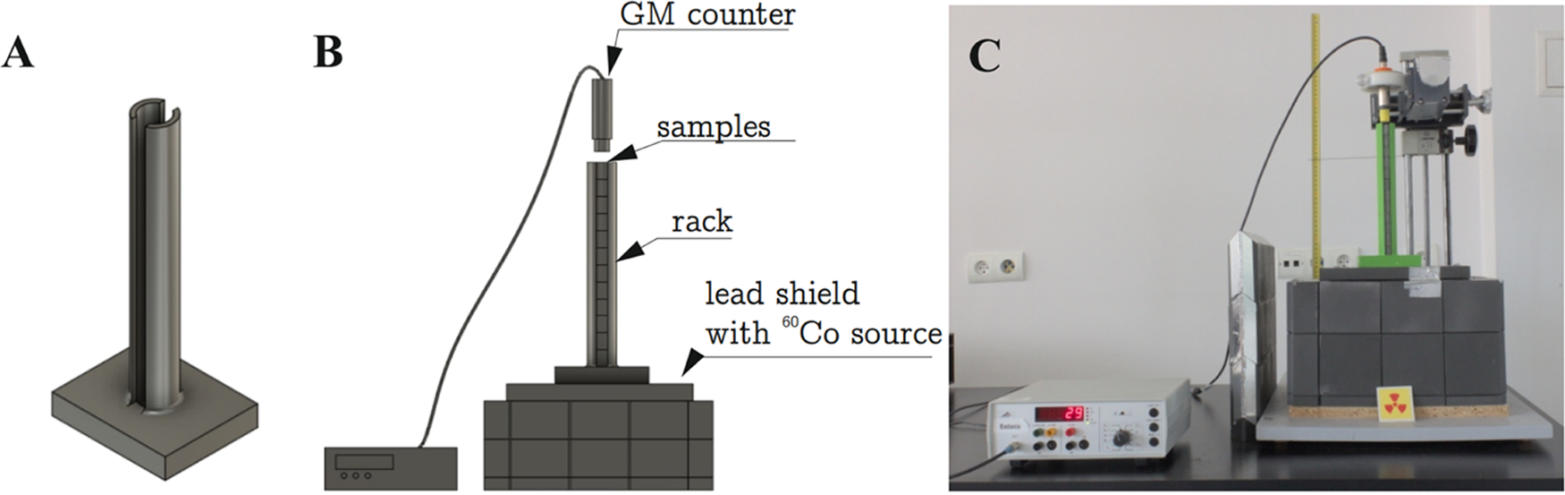
Isometric projection of the rack (A), a schematic picture of the setup for measuring the attenuation of γ radiation (B), and a photograph of the setup with the ^60^Co point source used in the study (C).

Each measuring series included fourteen (14) subsequent detections performed for the composite layer from 0 to 26 cm with a 2 cm step. Moreover, in order to ensure the stability of the added samples, a sample rack was designed (Figure 4A) and then manufactured using the fusion deposition method (FDM) on a 3D printer (Zortrax M300 Dual, Olsztyn, Poland). The experimental study of micro- and nanocomposites included three series of measurements. Each experimentally determined number of counts, *N*_exp_, was reduced by the determined background value, *N*_bg_, so that the presented result showed the photon counts coming from the source as *N*_reduced_ = *N*_exp_ – *N*_bg_.

### X-ray Shielding Measurements

The X-ray shielding measurements of manufactured nano- and microcomposites were carried out using a clinical X-ray diagnostic system type: PROTEUS, manufactured by GE Medical Systems. This system was equipped with a bifocal lamp with focal lengths of 0.6 and 1.2 mm. The total filtration of an X-ray tube and a collimator was 3.2 mm Al. The test was carried out for voltages from 70 to 130 kV in steps of 10 kV. The current–time load was at the level of 3.2 mAs. A digital detector, manufactured by AGFA (Mortsel, Belgium), characterized by sensitivity in the tested voltage range, had a pixel size of max 0.150 mm, an effective detector size of 2336 pixels × 2836 pixels, and a resolution of 3.36 lp mm^–1^.

The measurement methodology consisted of recording images of the tested samples for various anode voltages and a constant current–time load. The recorded images were saved according to the DICOM standard. It should be emphasized that the obtained images were not subjected to postprocessing, as this could affect the obtained calculation results. Numerical values, recorded by the detector and corresponding to the intensity of the X-ray radiation reaching the detector after passing through the sample, were used for the calculations. In each of the two sets of samples marked, one field was empty, and the other field was filled with paraffin of a thickness corresponding to the tested sample.

In order to carry out the calculations, it was necessary to read the digital values within the individual fields using MATLAB software (produced by The MathWorks, Portola Valley). For the analysis of correctness, it was necessary that the values taken to calculate the averages were from the same region of each sample. As indicated by the tested samples, the effect of air entrainment could be burdened with an increased error if the ROI did not come from exactly the same area. The averaging area covered a square with a side of 60 pixels × 60 pixels, which corresponds to an average value of 3600 pixels. The attenuation value was determined as the ratio of the intensity of the signal derived from the particular thickness of the sample layer *I*_layer sample_ to the intensity recorded in the aerated field *I*_air_, as the following eq 1 depicts
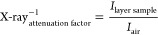
1

**Figure 5:**
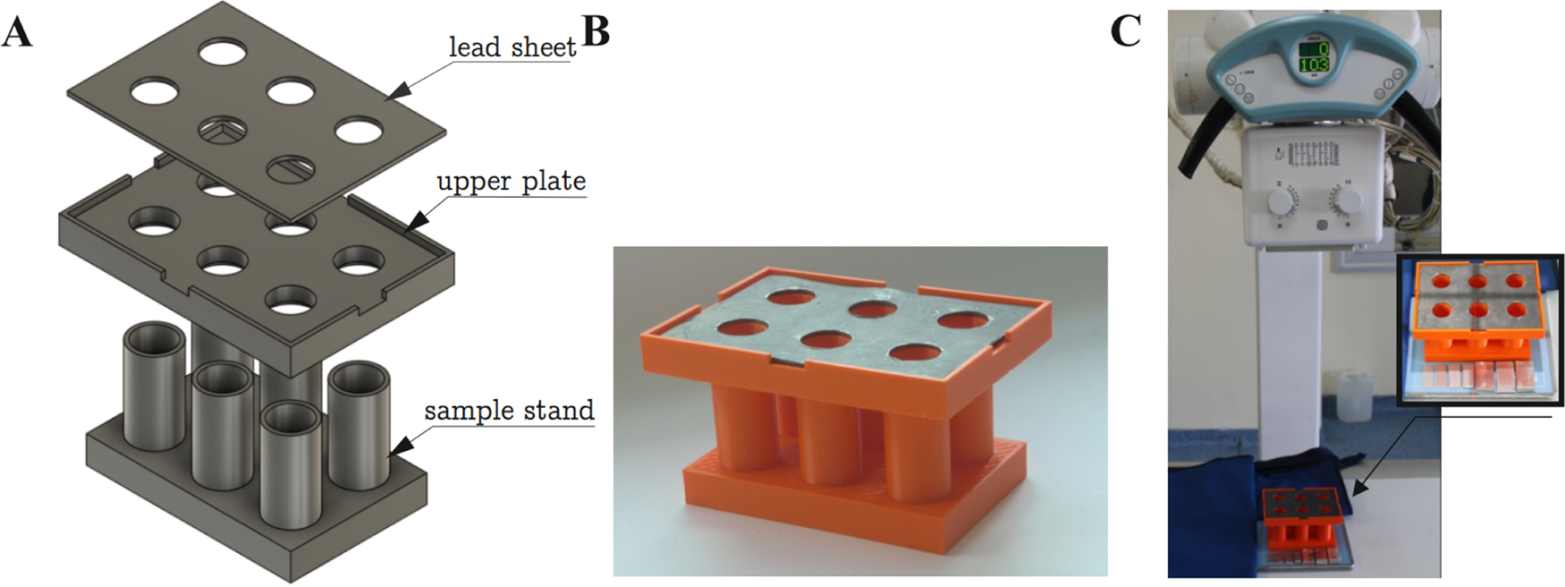
Isometric projection of the sample stand, with the upper plate and the lead sheet (A), the picture of the 3D-printed setup for measuring the attenuation of X-radiation (B), and the picture of an X-ray tube together with a sample setup (C).

Moreover, in order to ensure the stability of individual layer thicknesses of micro- and nanocomposites, a sample stand was designed for X-ray shielding measurements. In addition, a step to narrow the area down to the surface area of the micro- and nanocomposite discs was taken. Hence, an upper plate was designed, on which a 2 mm thick lead sheet with compatible holes was placed. Both the sample stand and the upper plate were manufactured by using FDM on a 3D printer (Zortrax M300 Dual, Olsztyn, Poland). The concept of the sample stand with the upper addition is shown in Figure 5A; the 3D printer-manufactured parts are shown in Figure 5B, and the whole set together with the X-ray tube are demonstrated in Figure 5C. In order to compare the damping capacity of individual nano- and microcomposites, aluminum plates with different layer thicknesses were placed next to the stand: 1, 2, 4, 6, 8, and 10 mm.

## Results and Discussion

**Figure 6:**
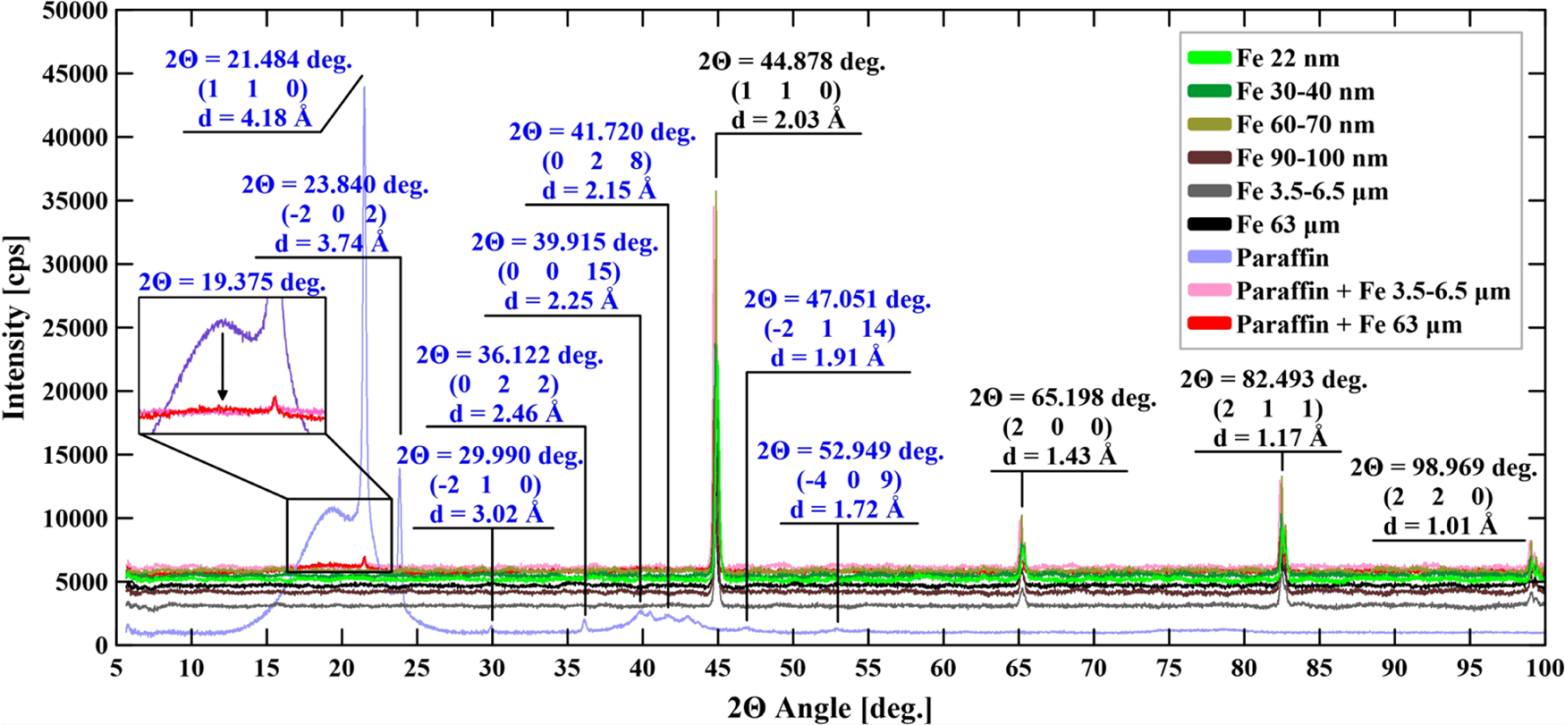
XRD spectra for Fe nanoparticles, pure paraffin, and the selected composite (black, peaks for Fe; blue, peaks for paraffin).

XRD spectra of the target composites are listed in Figure 6. The observed peaks were found to be in very good agreement with pattern 00–006–0696 for Fe and pattern 00–013–0675 for paraffin wax (the details are given in the Supporting Information in the S2 Section). A small peak at 2θ observed for the pure paraffin at 2θ = 19.375° is not observed for paraffin wax (see the Supporting Information, Table S12 and Figure S3); however, it occurs for the paraffins, for example, on patterns: 00–003–0259, 00–003–0254, and 00–014–0763. This behavior suggests that the paraffin used herein is in fact a mixture of a few compounds (see below for the results of NMR), in which one compound dominates.

To verify the composition of the paraffin wax, we used NMR spectroscopy. ^1^H and ^13^C NMR spectra (Figures S4–S7, respectively) of the neat paraffin (CDCl_3_, tetramethylsilane, TMS, as the reference compound) revealed characteristics typical for the linear (nonbranched) and symmetrical *n*-alkanes. In the ^1^H NMR spectra, the two main peaks could be found, i.e., at 0.88 and 1.25 ppm, corresponding to the terminal CH_3_ and the from-neighboring-to-middle chain CH_2_ groups, respectively. Quantification of the signals allowed us to confirm the average *n* number in the C*_n_*H_2*n*+2_ formula – oscillating around 34 (*n*-teatriacontane). The structure of the *n*-alkane skeleton was further confirmed in the ^13^C NMR spectra, where single peaks at 31.94, 29.72, 39.38, 22.71, and 14.13 ppm were found assignable to the following carbon atoms: C3, C5-middle, C4, C2, and C1, respectively.^28^

**Figure 7:**
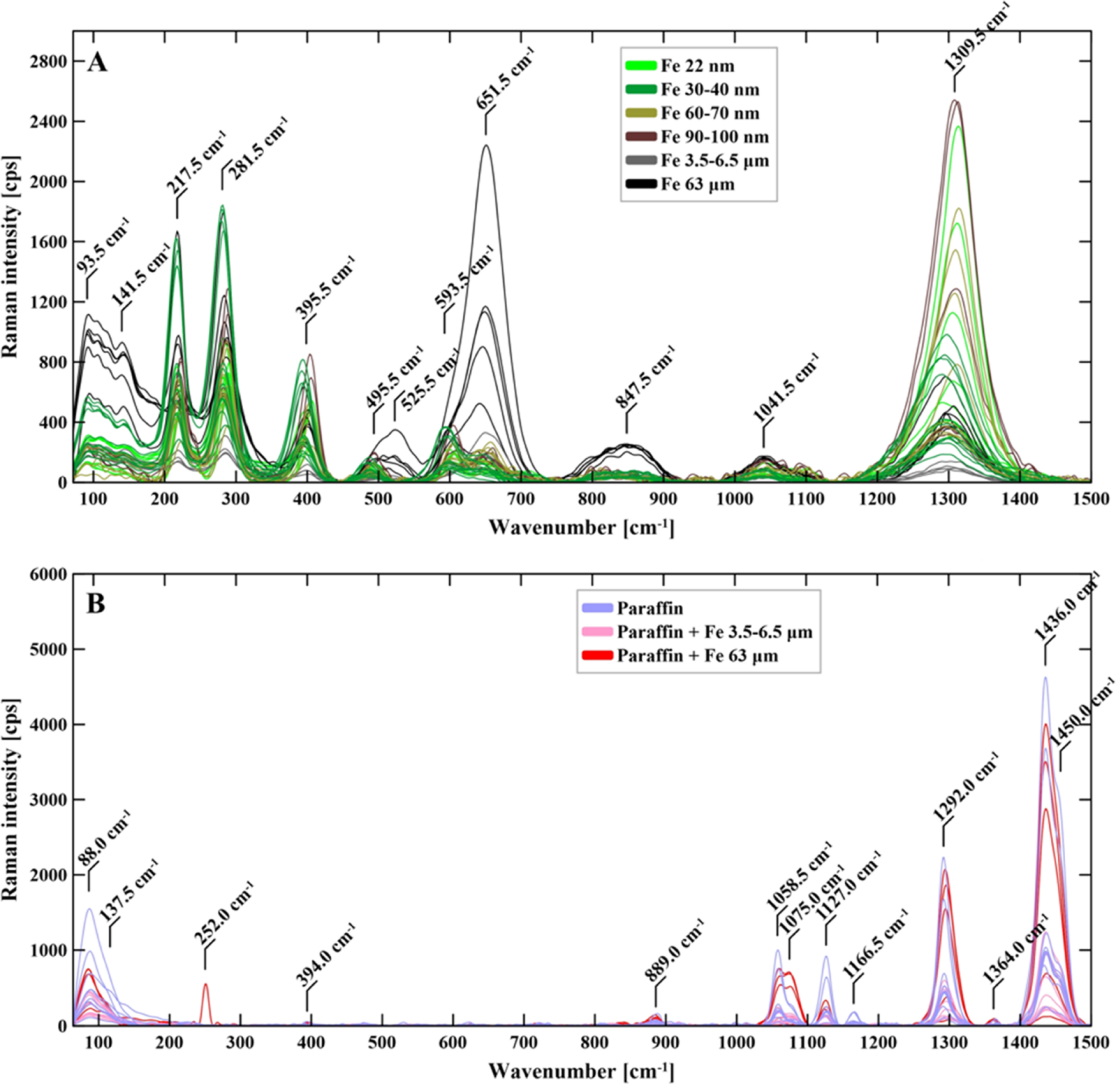
Raman spectra for the studied samples: Fe nano- and microparticles (A) and paraffin and the exemplary composite (B).

To examine the Fe–paraffin interactions and to determine the chemical form of the nanoparticles, we used Raman spectroscopy (Figure 7).

Since purely metallic phases do not show the polarizability change upon the acquisition of Raman spectra, due to a significantly higher specific surface area of the nano- and microsized nanoparticles, and hence the increased reactivity against the environmental oxygen and water vapor, the presence of surface-bound α-Fe_2_O_3_ phases could emerge as rather inevitable (Figure 7A). Indeed, the presence of superficial α-Fe_2_O_3_ was confirmed inter alia by the following peaks, with the most prominent ones at 225 (A_1g_), 291 (E_g_), 411 (E_g_), 499 (A_1g_), and 613 (E_g_) cm^–1^.^29^

Raman spectra of the neat paraffin (Figure 7B) revealed several peaks typical for *n*-alkanes, i.e., 1452, 1438, 1296, 1166, 1130, 1061, and 890 cm^–1^ corresponding to asymmetric bending δ(CH_3_), δ(CH_2_), wagging ν(C–C), antisymmetric stretching ν_as_(C–C), symmetric stretching ν_s_(C–C), and rocking ρ(CH_2_), respectively.^30,31^

The Raman spectra of the paraffin nano- and microcomposites (Figure 7B) were found to be enriched with signals derived from the α-Fe_2_O_3_ phase. Nevertheless, the neat paraffin signals remained intact (in terms of the shift location) within the range of statistical significance, revealing the interactions of paraffin and Fe/Fe_2_O_3_ as noncovalent. Thermal analysis was used mainly to investigate the stability of the composites. TGA of neat paraffin (Figure S8), both pyrolytic under nitrogen and combustion under air, revealed that the thermal behavior of the paraffin matrix was characterized primarily by volatilization gradually progressing from ca. 200 °C, with the maximum rate occurring at ca. 380 °C. At 800 °C, the gasification of paraffin was practically complete, while combustion accelerated the gasification, as the residue at 700 °C was equal to zero. TGA, again performed under both the pyrolytic (complete volatilization of paraffin and presence of intact iron) and combustional (burning of the paraffin matrix and oxidation of Fe to Fe_2_O_3_) regimes for all of the nano- and microcomposites, confirmed that within the statistical significance, the component contents were in full accordance with the presumed compositions of the manufactured composites. This fact corroborates the SEM/EDX results (see below).

**Figure 8:**
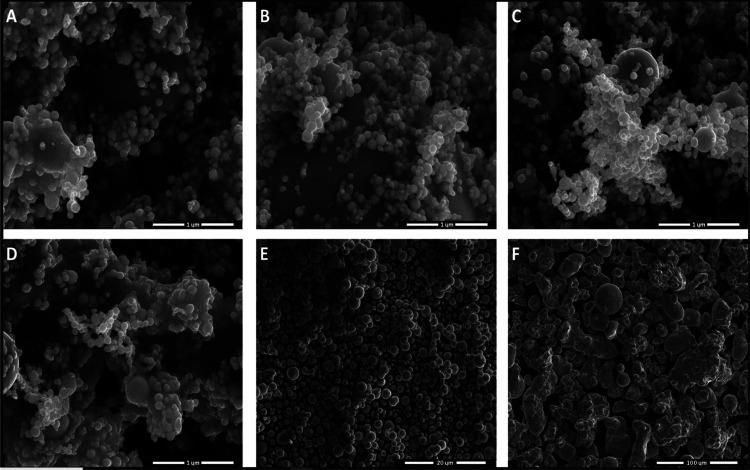
Selected SEM images of dry iron nanoparticles in sizes: 22 nm (A), 30–40 nm (B), 60–70 nm (C), 90–100 nm (D), 3.5–6.5 μm (E), and 63 μm (F).

Based on a series of SEM images, the size and shape of the Fe nano- and microparticles can be estimated. Figure 8 presents exemplary SEM images of the iron particles.

It can be observed that not all of the sample sizes are similar to those provided by the producers. Thus, the sample from Figure 8A is composed of the nanoparticles distributed in the range 40–140 nm, the sample from Figure 8B in the range 50–130 nm, the sample from Figure 8C in the range 30–130 nm, the sample from Figure 8D in the range 20–120 nm, the sample from Figure 8E in the range 0.25–3.5 μm, and finally, for the sample from Figure 8F, the microparticles are in the range 2.5–90 μm. Also, larger aggregates and grains are present in small amounts in all of the studied samples.

**Figure 9:**
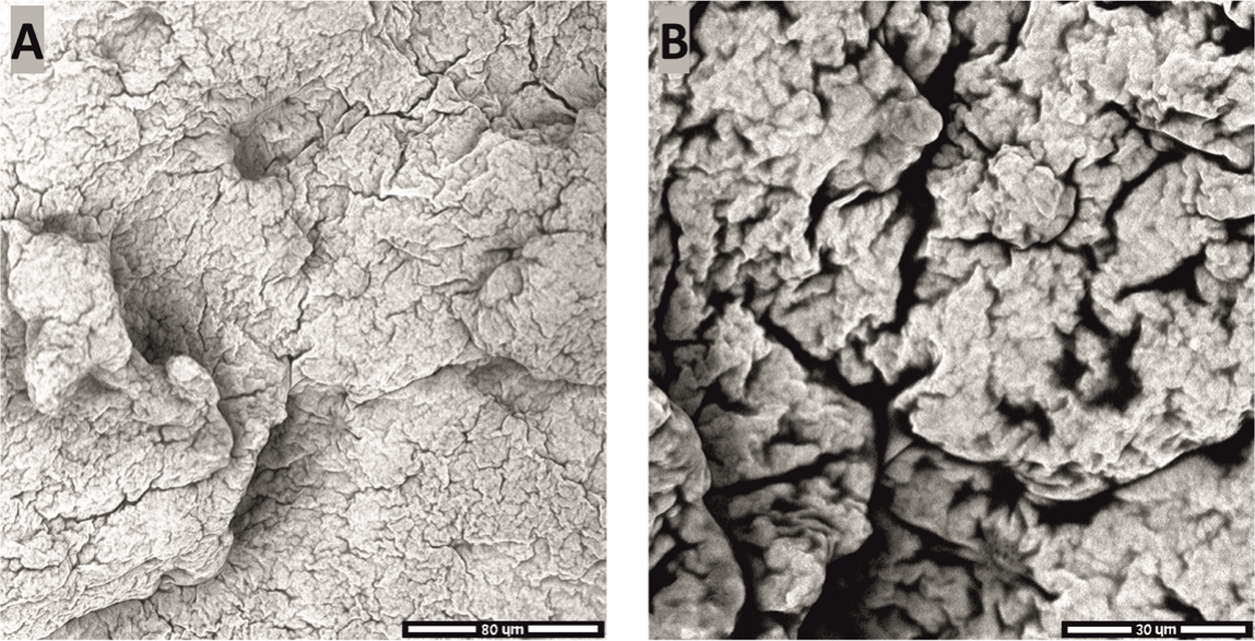
SEM images of pure paraffin at two magnifications: 1000× (A) and 2500× (B).

EDX results collected in the Supporting Information (Figures S9 and S10) confirm the presence of mainly iron on the surface with a smaller amount of oxygen and carbon. Furthermore, SEM images of the paraffin surface, in line with the observations described above, are depicted in Figure 9.

**Figure 10:**
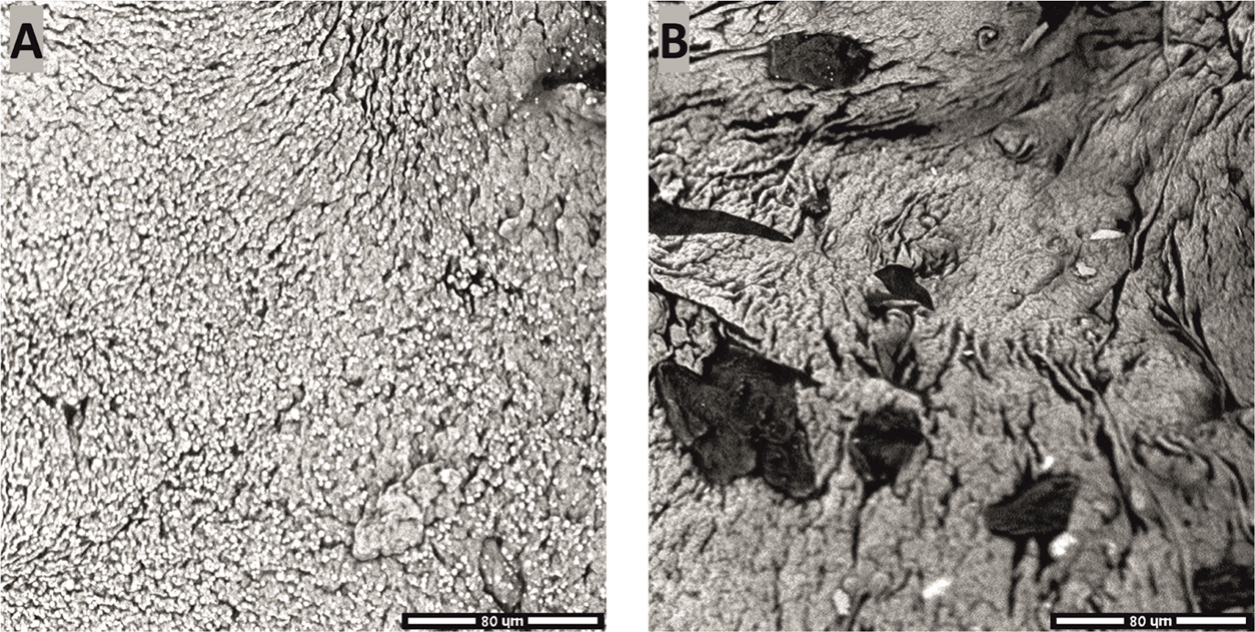
SEM images of paraffin-based microcomposites with 50 wt % iron addition: Fe 3.5–6.5 μm (A) and Fe 63 μm (B).

The collected images present a slightly cracked, semiporous surface with rounded and curved edges. Paraffin, used in this study, is plasticizable at room temperature; therefore, due to its susceptibility to a shape change upon, for instance, kneading, its surface reveals discontinuities enabling such a moldability. Additionally, porosity was further found as the feature corresponding to the adsorption of air though completely removable upon the manufacturing of the composites by heat treatment (above the melting point) and stirring. The manufactured composites (see Figure 10) had evenly distributed iron particles, which is confirmed by SEM. Furthermore, the smaller Fe particles emerged as more spheroidal and more homogeneously distributed through the paraffin matrix than their larger counterparts (visible also as more contrastive than the smaller ones).

### γ Shielding Ability

The shielding capabilities against ionizing radiation have been tested in two aspects. In the first stage, the influence of the particle size on the linear attenuation coefficient was determined. In the second stage, tests were carried out to determine the effect of the concentration of iron particles in the composite on the shielding properties of the composite. The results of these studies are presented below.

### Size Dependence

Selecting a proper shield against ionizing radiation requires knowledge about its attenuating ability; therefore, one of the factors considered in this aspect is the linear attenuation coefficient μ (cm^–1^). It is a constant, describing the probability of photon interaction per unit of linear path in the shielding material,^32,33^ and it is presented in a well-known dependence of the radiation attenuation by shields, expressed by the following equation
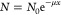
2where *N* and *N*_0_ are the counts for the shielding material and the initial number coming directly from the source, respectively, and *x* is the thickness of the shielding material. Based on the obtained results, the μ coefficients were calculated for pure paraffin and each of the composites and were developed by fitting an exponential curve to experimental data with the equation modeled as presented in eq 3
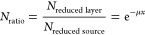
3where *N*_ratio_ is the ratio of the counts collected after the layer was applied, *N*_reduced layer_, to the value obtained without any layer, *N*_reduced source_, while μ is the linear attenuation coefficient (cm^–1^), and *x* is the material layer thickness. The experimentally obtained values of *N*_ratio_ decrease along with the increasing thickness of micro and nanocomposites, as summarized in Table S13 in the Supporting Information. Table 2 contains the values of the determined linear attenuation coefficients μ of the manufactured composites.

**Table 2:** Summary of Experimentally Designated Linear Attenuation Coefficient μ (cm^–1^) with Uncertainty and Determined Half Value Layer HVL (cm) for Different Micro- and Nanocomposites

sample	linear attenuation coefficient μ (cm^–1^)	HVL (cm)
Paraffin	0.04550 ± 0.00047	15.23 ± 0.22
Paraffin + 10 wt % Fe 22 nm	0.05015 ± 0.00077	13.82 ± 0.31
Paraffin + 10 wt % Fe 30–40 nm	0.05187 ± 0.00049	13.36 ± 0.18
Paraffin + 10 wt % Fe 60–70 nm	0.05258 ± 0.00083	13.18 ± 0.30
Paraffin + 10 wt % Fe 90–100 nm	0.05005 ± 0.00053	13.85 ± 0.21
Paraffin + 10 wt % Fe 3.5–6.5 μm	0.04918 ± 0.00039	14.09 ± 0.16
Paraffin + 10 wt % Fe 63 μm	0.05064 ± 0.00088	13.68 ± 0.34

The results show that, compared to the pure paraffin, nano- and microcomposites containing 10 wt % iron particles displayed an enhanced shielding performance, reaching a higher μ coefficient by about 10.22, 14, 15.56, 10, 8.09, and 11.30% for the composites containing iron particles of 22 nm, 30–40 nm, 60–70 nm, 90–100 nm, 3.5–6.5 μm, and 63 μm particle size, respectively.

On the other hand, it should be noted that attenuation of the nano- and microcomposites with 10 wt % addition of iron particles is similar; although the composites contained different sizes of iron particles (varying from 22 nm to 63 μm), the effect of the diameter of the iron particle size on the attenuation capacity was not visible.

**Figure 11:**
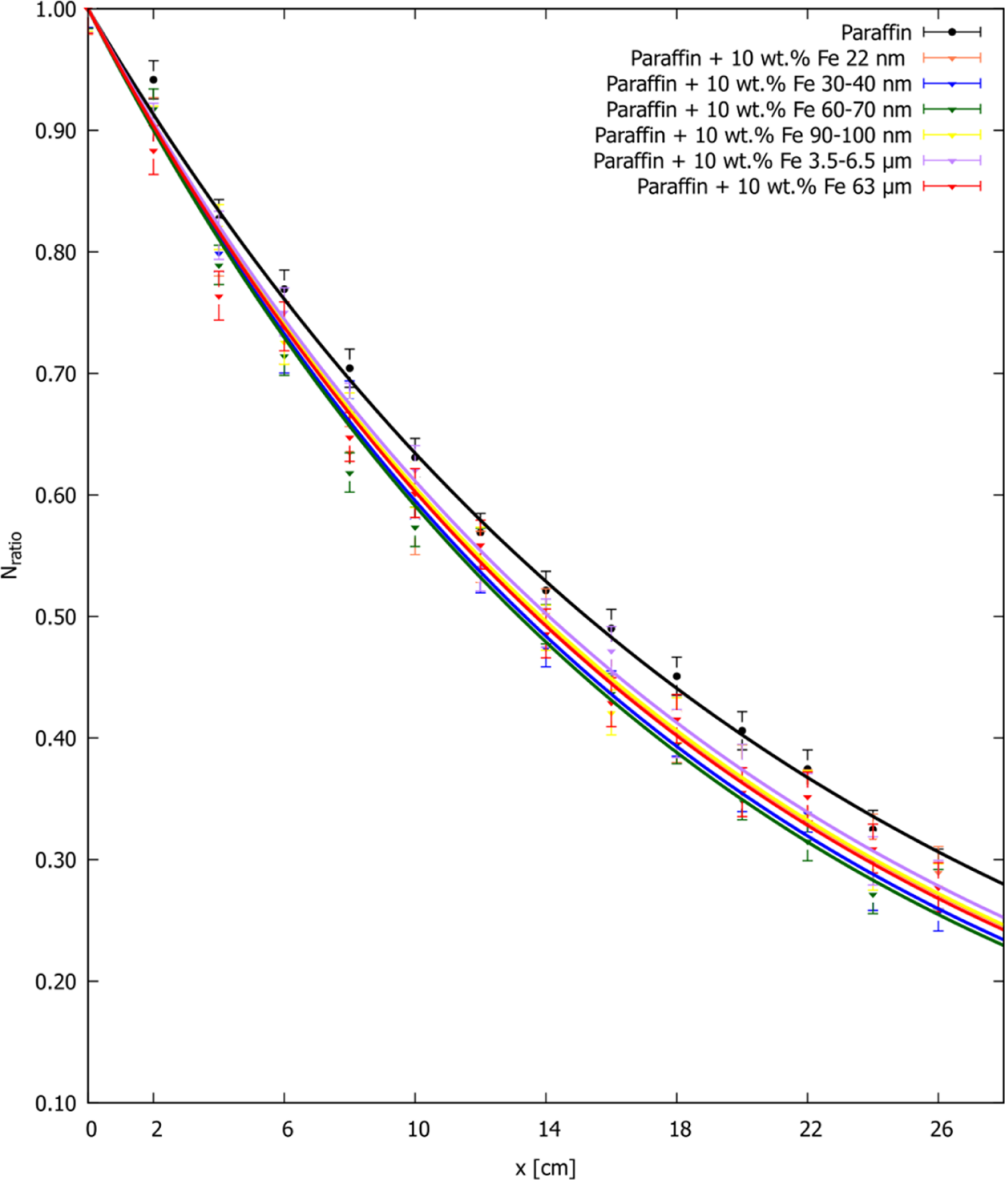
Dependence of the ratio of the number of detected photons for each examined layer thickness to the number of photons collected without any layer on the thickness of the composite with 10 wt % addition of Fe 22 nm, Fe 30–40 nm, Fe 60–70 nm, Fe 90–100 nm, Fe 3.5–6.5 μm, and Fe 63 μm iron particles. The symbols and lines represent the experimental and eq 3 fitting data, respectively.

The highest μ value was recorded for the nanocomposite containing 10 wt % Fe 60–70 nm particles, thus confirming the most efficient attenuation compared to the other composite samples; however, all micro- and nanoparticle linear attenuation coefficients oscillate around 0.05 cm^–1^. This behavior could be illustrated by Figure 11, where a nearness in the shielding ability of the individual nanocomposites can be seen. On the other hand, the characteristics of suppression for the individual cases in the form of single graphs presenting the ratio of *N*_ratio_ to the thickness *x* of the individual layers of nano- and microcomposites are shown in Figure S11 in the Supporting Information.

Considering the practical application of the shielding material, the more important parameter is its half value layer (HVL). The lower HVL of the shielding material contributes to the smaller amount of used raw materials and the lower financial outlay. The physical meaning of this parameter is the determination of the layer thickness of the shielding material, after which the initial intensity of radiation decreases by 50%. To determine its value, it is necessary to know the linear attenuation coefficient, so it could be calculated as
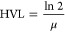
4

The HVL values were calculated and are presented in Table 2.

**Figure 12:**
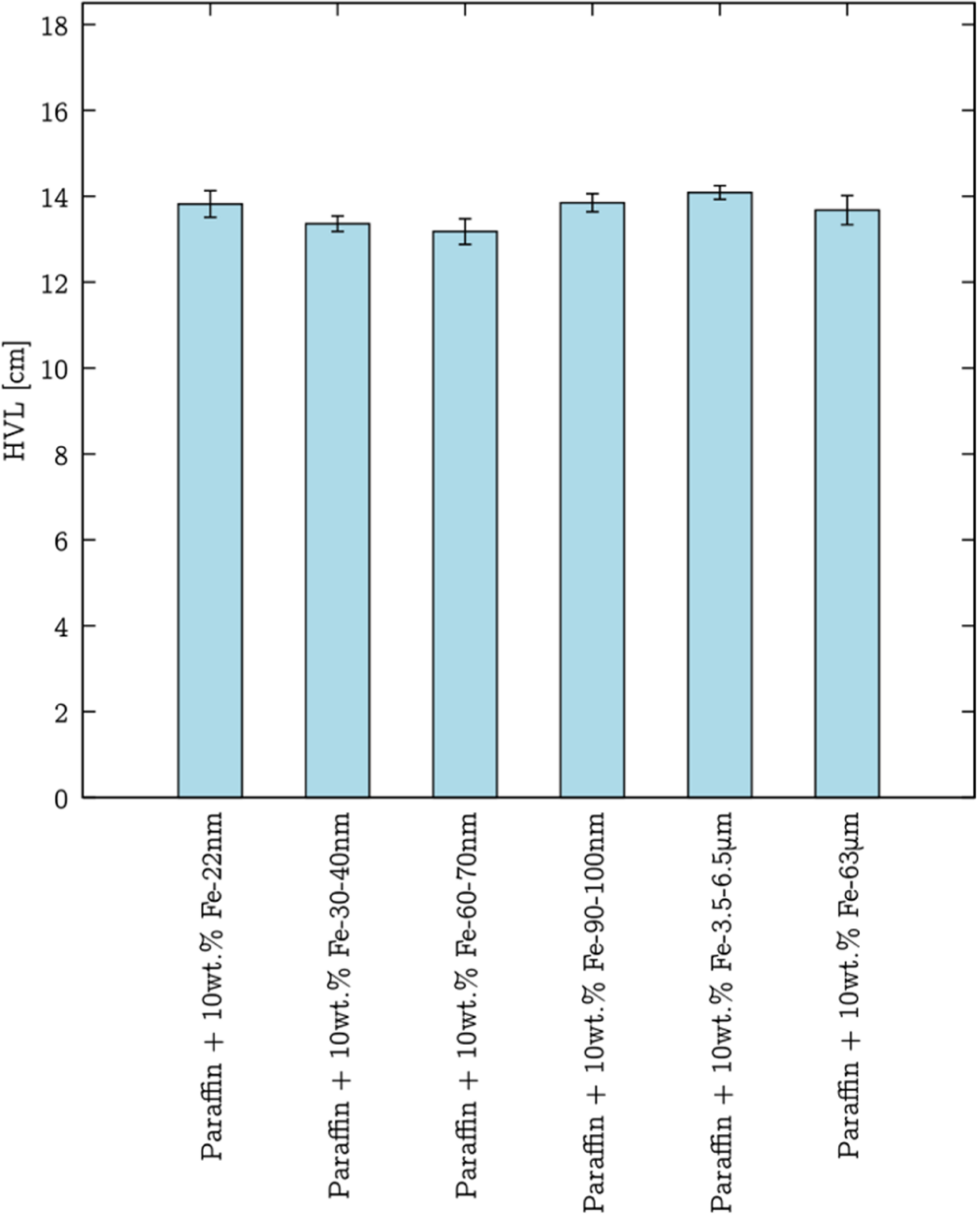
Half value layer HVL (cm) for the nano- and microcomposites filled with 10 wt % iron particles, different in size (shown as individual bars). The precise HVL values, together with uncertainties, are given in Table 2.

Again, it can be noticed that although the HVL values of nano- and microcomposite samples differ, they are within 14 cm of thickness, which is also confirmed by the histogram in Figure 12. Compared to the value of paraffin, the half-thickness values of composites with the 10 wt % addition of Fe 22 nm, Fe 30–40 nm, Fe 60–70 nm, Fe 90–100 nm, Fe 3.5–6.5 μm, and Fe 63 μm are 9.26, 12.28, 13.46, 9.06, 7.49, and 10.18% lower, respectively.

### Concentration Dependence

**Figure 13:**
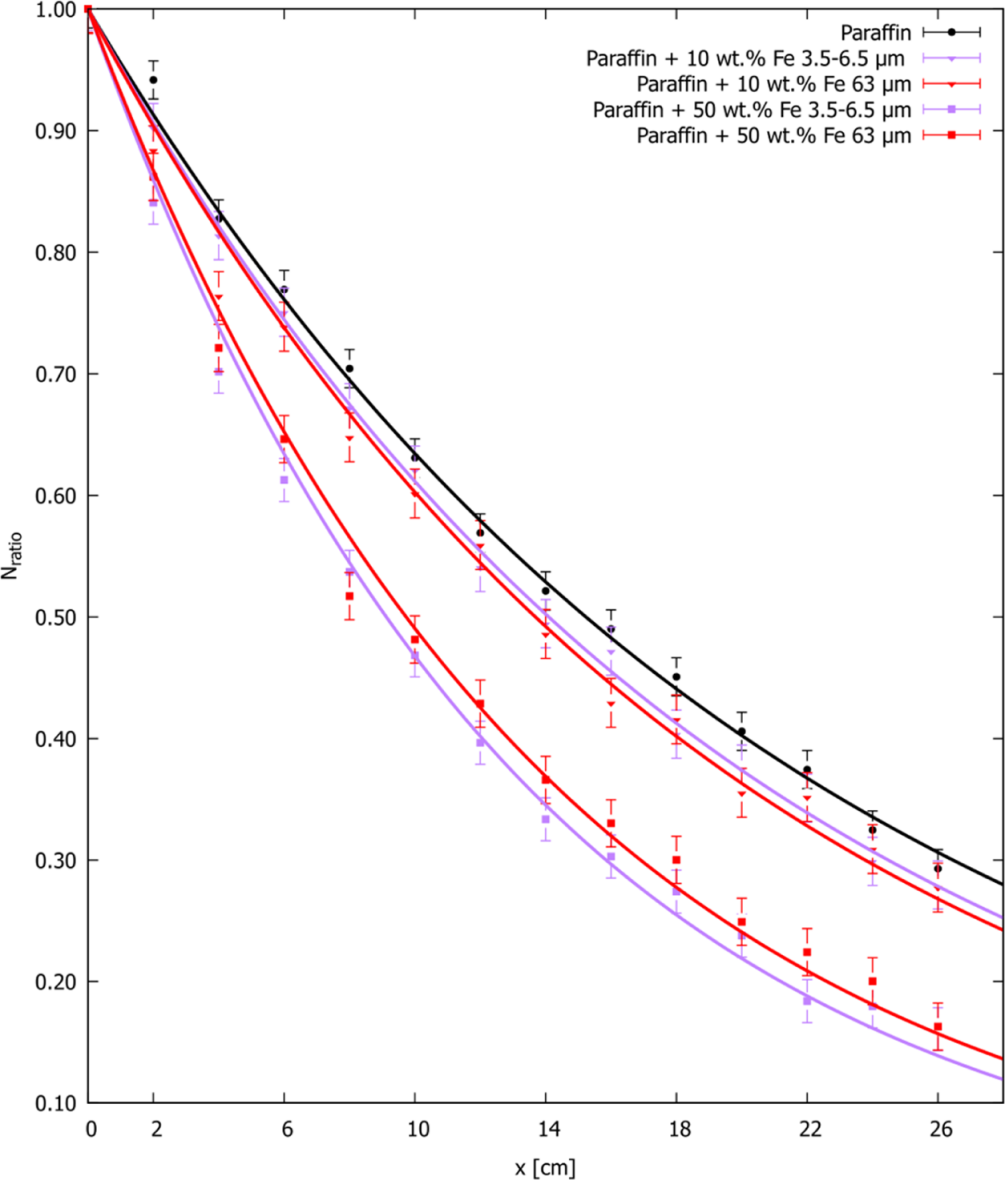
Dependence of the ratio of the number of detected photons for each examined layer thickness to the number of photons collected without any layer on the thickness of the composite with 10 and 50 wt % addition of Fe 3.5–6.5 μm and Fe 63 μm microparticles. Symbols present experimental data; lines present eq 3 fitting.

The shielding properties of microcomposites developed in this study were evaluated for composites containing two different mass concentrations of particles. The results of those measurements are presented in Figure 13 and in Table S14 in the Supporting Information. The list of μ and HVL values was calculated and is summarized in Table 3.

**Table 3:** Summary of Experimentally Designated Linear Attenuation Coefficient μ (cm^–1^) with Uncertainty and Determined Half Value Layer HVL (cm) for Different Microcomposites

sample	linear attenuation coefficient μ (cm^–1^)	HVL (cm)
Paraffin	0.04550 ± 0.00047	15.23 ± 0.22
Paraffin + 10 wt % Fe 3.5–6.5 μm	0.04918 ± 0.00039	14.09 ± 0.16
Paraffin + 10 wt % Fe 63 μm	0.05064 ± 0.00088	13.68 ± 0.34
Paraffin + 50 wt % Fe 3.5–6.5 μm	0.0760 ± 0.0012	9.12 ± 0.21
Paraffin + 50 wt % Fe 63 μm	0.0712 ± 0.0012	9.74 ± 0.24

Based on the obtained data, it is clear that the linear attenuation coefficient of composites increased with a higher iron particle content. Accordingly, including 10 and 50 wt % Fe 3.5–6.5 μm microparticles, the shielding ability of the composites was enhanced by ca. 8 and 67%, respectively, in comparison to the pure paraffin. Considering HVL values, a fact can also be noticed: a thickness of about 15 cm of paraffin reduces the number of counts from the source by half, while in the case of both microcomposites with 50 wt.% iron particles, it is around 9 cm.

Based on this, it can be stated that the increase in the content of microsized matter improves its protective properties. The uniform dispersion of iron particles allows for multiple scattering of the incident γ rays, and as a result, it leads to an overall improvement in the shielding properties. Moreover, the content of filler and its contribution to the improvement of the shielding properties of materials were presented earlier, see Refs (34−36) as an example. Especially, worth mentioning is Ref. (34), where authors studied isophthalic polyester (PES) as the main component with a natural microsized mineral (hematite) in 10, 20, 30, 40, and 50% addition by weight. The obtained mass attenuation coefficients μ_m_ (cm^2^ g^–1^) at 662 keV for the microcomposite with 50 wt % hematite achieved 98% of μ_m_ of the elemental lead value, being approximately 58% lighter at the same time.

According to the literature data, the linear attenuation coefficients of iron for 1.171 and 1.333 MeV energy values are 0.479 and 0.384 cm^–1^, respectively.^13^

Considering the iron attenuation ability designated for the ^60^Co source from the literature data, as well as studied microcomposites with 50 wt % Fe 3.5–6.5 μm and 50 wt % Fe 63 μm particles, the γ shielding properties of the manufactured samples are 84.13 and 85.15% lower, respectively, in comparison to the value of pure iron. The layer that will halve the number of counts for a ^60^Co source for pure iron is 1.45 cm (1.171 MeV) and 1.81 cm (1.333 MeV), and in the case of manufactured microcomposites with the highest iron content, HVL oscillates around 9 cm. Similarly, decent shielding properties can be achieved for the Fe_3_O_4_–Al–PVA nanocomposite,^13^ whose HVL value is lower than that of pure iron. HVL of other proposed shielding composites ranges from approximately 4 cm (for example, 3.82 cm for Ba–Fe–Ni oxide nanocomposite^9^) to approximately 6 cm for α-Fe_2_O_3_-HDPE-40 wt % nanocomposite.^12^

**Figure 14:**
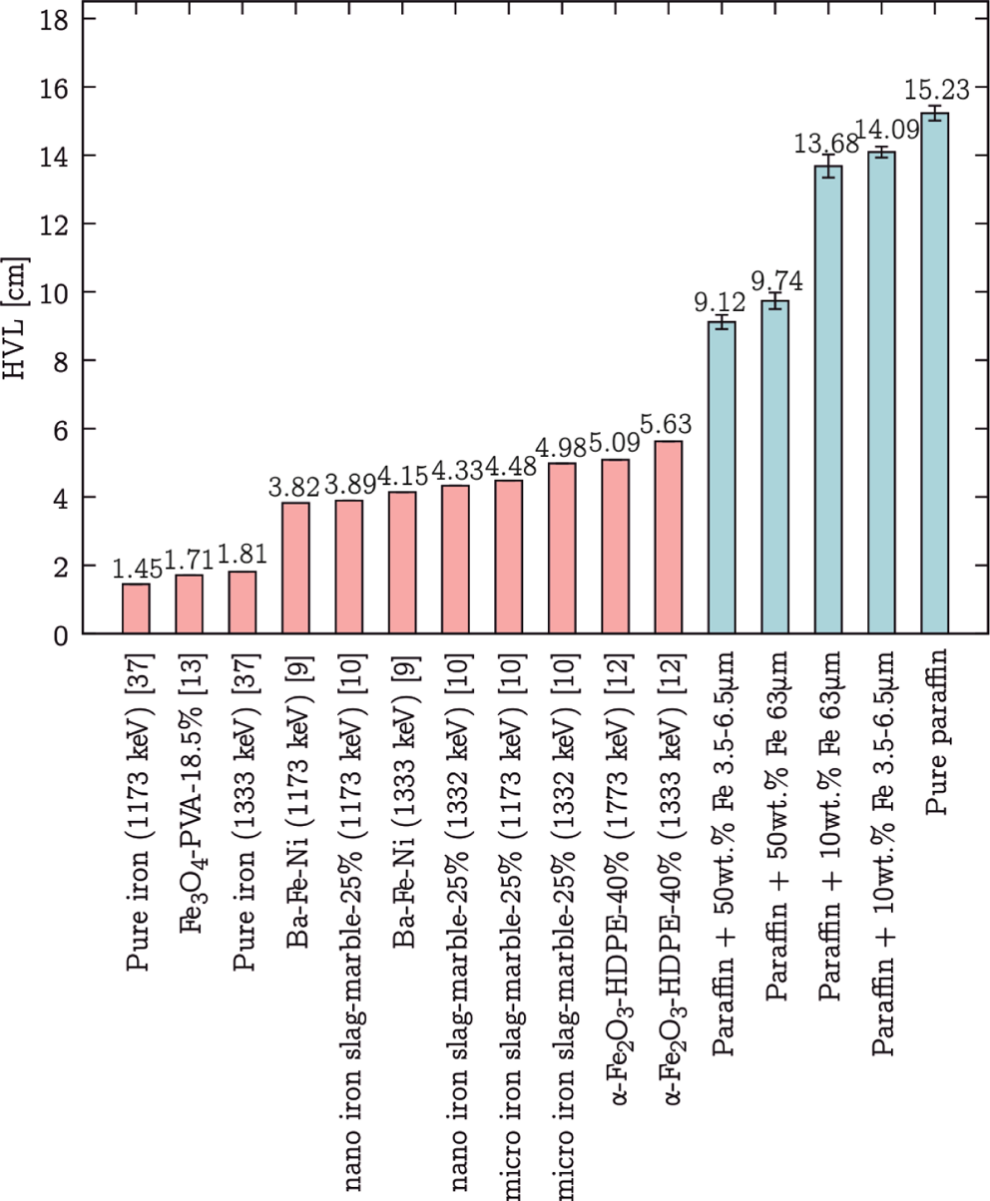
Half value layer HVL (cm) for the pure paraffin and microcomposites filled with 10 and 50 wt % iron particles, different in size 3.5–6.5 and 63 μm, compared to HVL of pure iron and other nano- and microcomposites in the literature. The precise HVL values, together with the uncertainties of the measured microcomposites and pure paraffin, are given in Table 3.

Although the more protective nature of pure iron and other proposed micro- and nanocomposites is indisputable in comparison to the microcomposites investigated in our study, their use is limited; therefore, a wider application can be envisaged for the easily moldable iron-doped microcomposites. A comparison of these values, together with HVL values of studied paraffin, and microcomposites, pure iron, and other manufactured nano- and microcomposites from literature data, can be particularly observed in the histogram in Figure 14. Moreover, the results of the HVL (cm) value and linear absorption coefficient μ (cm^–1^) were tabulated (Table S15).

### X-ray Shielding Ability

The X-ray shielding properties of manufactured nano- and microcomposites have been explored in two aspects, as in the case of γ radiation: iron particle size and their concentration. Although the X-ray measurement covered anode voltage values from 70 to 130 kV with a step of 10 kV, in the following section, the analysis was focused on 70 kV, because it is the average value of the anode voltage used in the X-ray diagnostics. Nevertheless, the results from the other voltage values are included in the Supporting Information in Figure S12, along with the summarized data in Tables S16–S29.

Generally, the intensity of the damped X-rays after passing through the material can be summarized in the exponential attenuation dependence, expressed as
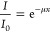
5where *I* and *I*_0_ are signal intensity after passing a shielding material and intensity without any layer of shielding material, respectively, while μ is the linear attenuation coefficient (cm^–1^) and *x* is the thickness of the shielding material. Focusing on our measurements, the μ coefficients were calculated for pure paraffine, aluminum plates, and each composite by fitting an exponential curve to experimental data at 70 kV, with the equation, modeled as below in eq 6
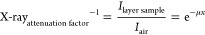
6where *I*_layer sample_ is the signal intensity coming from the sample layer, *I*_air_, to the intensity value of the aerated field, μ is the linear attenuation coefficient (cm^–1^), and *x* is the material layer thickness. The summarized linear attenuation coefficient μ (cm^–1^) for all nano- and microcomposites at a 70 kV anode voltage is presented in Table 4, together with calculated HVL values.

**Table 4:** Summary of Experimentally Designated Linear Attenuation Coefficient μ (cm^–1^) with Uncertainty and Determined Half Value Layer HVL (cm) for Different Micro- and Nanocomposites Containing 10 wt % Iron Particles and Aluminum at 70 kV Anode Voltage

sample	linear attenuation coefficient μ (cm^–1^)	HVL (cm)
paraffin	0.1795 ± 0.0020	3.861 ± 0.062
paraffin + 10 wt % Fe 22 nm	0.4179 ± 0.0066	1.659 ± 0.038
paraffin + 10 wt % Fe 30–40 nm	0.4280 ± 0.0104	1.619 ± 0.057
paraffin + 10 wt % Fe 60–70 nm	0.4351 ± 0.0139	1.593 ± 0.073
paraffin + 10 wt % Fe 90–100 nm	0.4372 ± 0.0173	1.585 ± 0.091
paraffin + 10 wt % Fe 3.5–6.5 μm	0.3683 ± 0.0089	1.882 ± 0.066
paraffin + 10 wt % Fe 63 μm	0.3790 ± 0.0058	1.829 ± 0.041
aluminum	0.1036 ± 0.0440	6.689 ± 0.411

**Figure 15:**
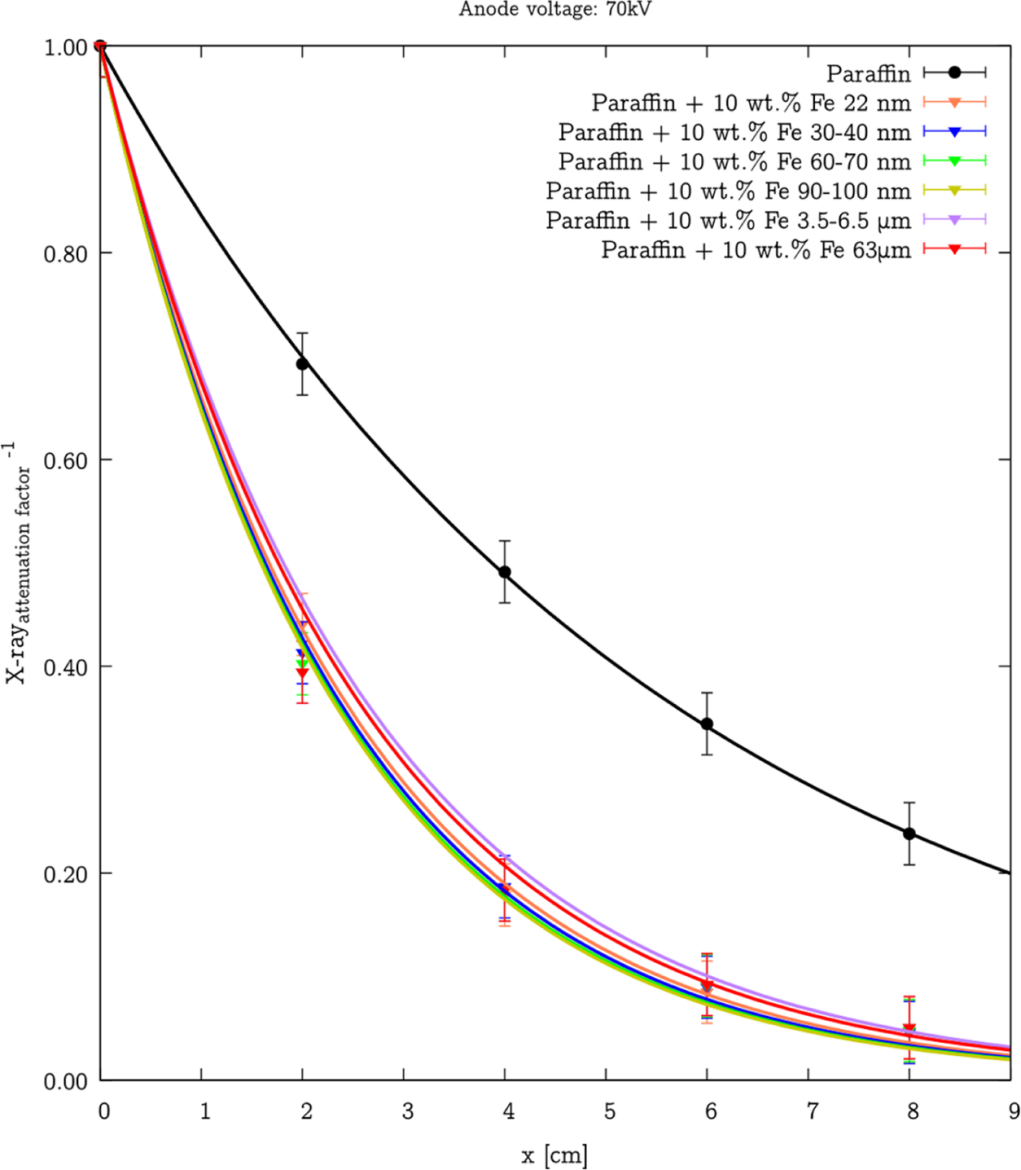
Dependence of the X-ray_attenuation factor_^–1^ for various thicknesses at 70 kV anode voltage for the nano- and microcomposites with 10% addition of Fe (22 nm), Fe (30–40 nm), Fe (60–70 nm), Fe (90–100 nm), Fe (3.5–6.5 μm), and Fe (63 μm) iron particles. Symbols present experimental data, and lines present eq 6 fitting. Considering the conditions of the X-ray measurements (radiography exposure time), the accuracy can be estimated at ±3%.

Based on the achieved data, it can be noticed that the ratio of the signal intensity of the examined sample layer thickness to the field aeration intensity decreased with the increase of the layer thickness, which is also numerically confirmed by the summary in Table S15 in the Supporting Information. The obtained outcomes revealed that nano- and microcomposites with 10 wt % iron particle additions displayed a superior attenuating ability in comparison to the pure paraffin. The composites were found to be more efficient attenuators than the pure paraffin by 32, 38, 42, 43, 5, and 11% for the samples filled with 10 wt % of 22 nm, 30–40 nm, 60–70 nm, 90–100 nm, 3.5–6.5 μm, and 63 μm iron particles at 70 kV. However, considering the particle size of the manufactured nano- and microcomposites, it can be concluded that even though paraffin composites with iron nanoparticles exhibited a higher X-ray attenuating ability, the effect is rather mediocre (Figure 15). Moreover, the obtained linear attenuation coefficients of nano- and microcomposites with 10 wt % iron particle addition are similar, oscillating from 0.36 to 0.43 cm^–1^.

Considering the HVL values, the lowest value was determined for the nanocomposite with 10 wt % iron particles with 90–100 nm size, which is 1.585 cm, being 58% lower than the HVL value for paraffin. Nanoparticles, due to their smaller size in comparison to particles in the microsize range (which may also form agglomerates), can be more dispersed in the paraffin volume, thus increasing the probability of interaction of the radiation beam with the iron nanoparticle, as shown in the achieved data. Moreover, enhanced X-ray shielding properties of the composites with only 10 wt % iron particles compared to the tested aluminum tiles were also discovered, which shows a relevant contribution to the field.

Analyzing the content of matter, it can be stated, similarly to the study of γ radiation attenuation, that a higher content of iron particles results in enhanced X-ray attenuation for the so-manufactured microcomposites. Again, it can be emphasized that more dispersed microparticles cause a probability increase in the interaction of the incident X-radiation with iron particles, causing the same X-ray beam attenuation. Considering the calculated linear attenuation coefficient (μ) for the microcomposite with 50 wt % iron particles 3.5–6.5 and 63 μm, they are 1.4987 and 1.4799 cm^–1^, accordingly, while for pure paraffin and aluminum plates, they are 0.1795 and 0.1036 cm^–1^, respectively, as summarized in Table 5.

**Table 5:** Summary of Experimentally Designated Linear Attenuation Coefficient μ (cm^–1^) with Uncertainty and Determined Half Value Layer HVL (cm) for Microcomposites Doped with 10 and 50 wt % Iron Microparticles and Aluminum at 70 kV Anode Voltage

sample	linear attenuation coefficient μ (cm^–1^)	HVL (cm)
paraffin + 10 wt % Fe 3.5–6.5 μm	0.3683 ± 0.0089	1.882 ± 0.066
paraffin + 10 wt % Fe 63 μm	0.3790 ± 0.0058	1.829 ± 0.041
paraffin + 50 wt % Fe 3.5–6.5 μm	1.4987 ± 0.0311	0.462 ± 0.014
paraffin + 50 wt % Fe 63 μm	1.4799 ± 0.0332	0.468 ± 0.015
aluminum	0.1036 ± 0.0044	6.689 ± 0.411

**Figure 16:**
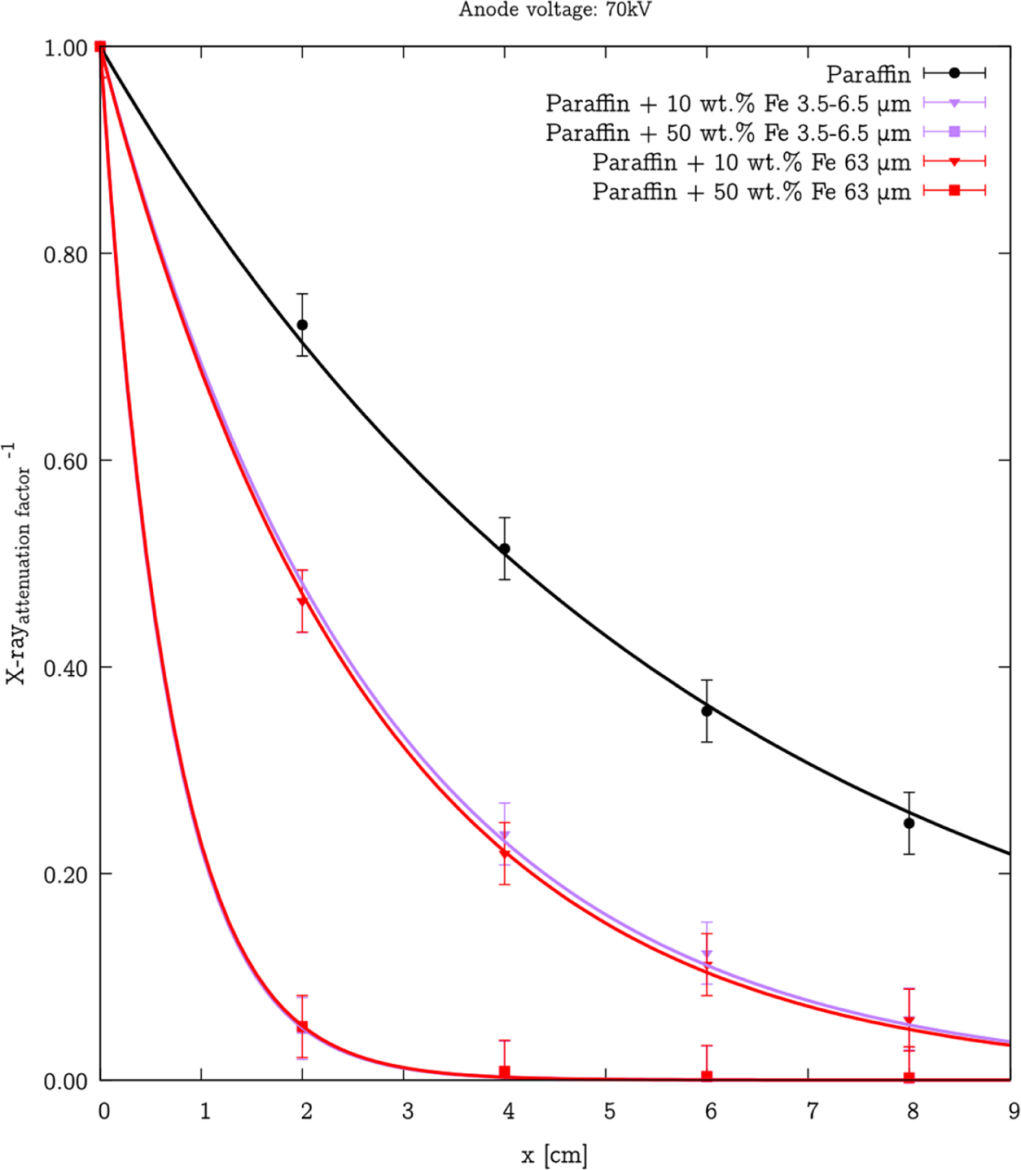
Dependence of the X-ray_attenuation factor_^–1^ for various thicknesses at 70 kV anode voltage for the microcomposites with 10 and 50 wt % addition of Fe 3.5–6.5 μm and Fe 63 μm iron particles. Symbols present experimental data, and lines present eq 6 fitting. Considering the conditions of the X-ray measurements (radiography exposure time), the accuracy can be estimated at ±3%.

From a more practical perspective, i.e., the material thickness that attenuates half of the incident radiation, after comparing the values for pure paraffin (3.861 cm) and composite with 50 wt % Fe 63 μm (1.829 cm), it can be noticed that higher contents of the microparticles improve the X-ray shielding parameters of microcomposites. Figure 16 shows the dependence of the X-ray_attenuation factor_^–1^ for the microcomposites with 10 and 50 wt % addition of Fe 3.5–6.5 μm and Fe 63 μm iron particles, confirming the advantageous attenuating ability for microcomposites with higher iron particle content.

The video showing the practicality in shape changing of the manufactured shielding material with the warmth of the hand is added in the Supporting Information (see Section S7a)

Considering γ-radiation, we found that values of the linear attenuation coefficient of paraffin-based micro- and nanocomposites containing 10 wt % iron particles oscillated around 0.05 cm^–1^; however, compared to the pure paraffin, their shielding ability is 10.22, 14, 15.56, 10.00, 8.09, and 11.30% higher for the composites containing Fe 22 nm and Fe 30–40 nm, Fe 60–70 nm, Fe 90–100 nm Fe 3.5–6.5 μm, and Fe 63 μm particles, respectively.

The subject of the influence of particle size on shielding properties has already been raised in the literature.^37−40^ We also found that nanoparticles had higher shielding efficiency and protection compared to microparticles, especially at lower γ-ray energies. Undoubtedly, the smaller nanoparticles allowed for better dispersion in the main composition, thus making beam–particle interactions more likely.^39^ Therefore, it could be concluded that smaller additives had an advantageous effect on the shielding ability and the enhanced dispersibility, while this influence is not significant, probably due to the effective size of the nanoparticle agglomerates embedded in the matrix. Similarly, the results confirm that increasing the content of the micro- and nanoparticles appreciably improves the shielding abilities of the manufactured composites. Here, it was also proven that the increasing iron particle content affected the improvement of the γ-ray shielding ability. As compared to the pure paraffin, the composites doped with 50 wt % Fe 3.5–6.5 μm exhibited a higher γ-attenuation ability by 67%, while 50 wt % Fe 63 μm by 56%. This is also reflected in the half-thickness: in the case of pure paraffin, a thickness of 15 cm attenuates half of the number of counts (*N*_reduced layer_), while in the case of a composite with 50 wt% iron micromatter, it is 9.12 and 9.74 cm for Fe 3.5–6.5 μm and Fe 63 μm, respectively.

In the case of X-rays (focusing only on 70 kV anode voltage), the linear attenuation coefficients μ of paraffin-based micro- and nanocomposites containing 10 wt % iron particles were also similar, around 0.4 cm^–1^. It should also be emphasized that the difference in the sizes of the added iron particles in the composites did not translate into a significant change in the parameters of the protective properties. Interestingly, composites with only 10% of iron nano- and microparticles are more efficient shields than aluminum plates (μ = 0.10 cm^–1^).

As in the case of the γ shielding measurements, a higher content of iron particles significantly improved the X-ray shielding properties. This phenomenon is related to the higher probability of the interaction of the incident radiation with iron particles. Focusing on the half value layer, thicknesses of ca. 4, 6, and 0.5 cm caused attenuation of half of the X-ray incident beam for pure paraffin, aluminum plates, and the composite with 50 wt % iron microparticles, respectively.

### Mechanistic Considerations

**Figure 17:**
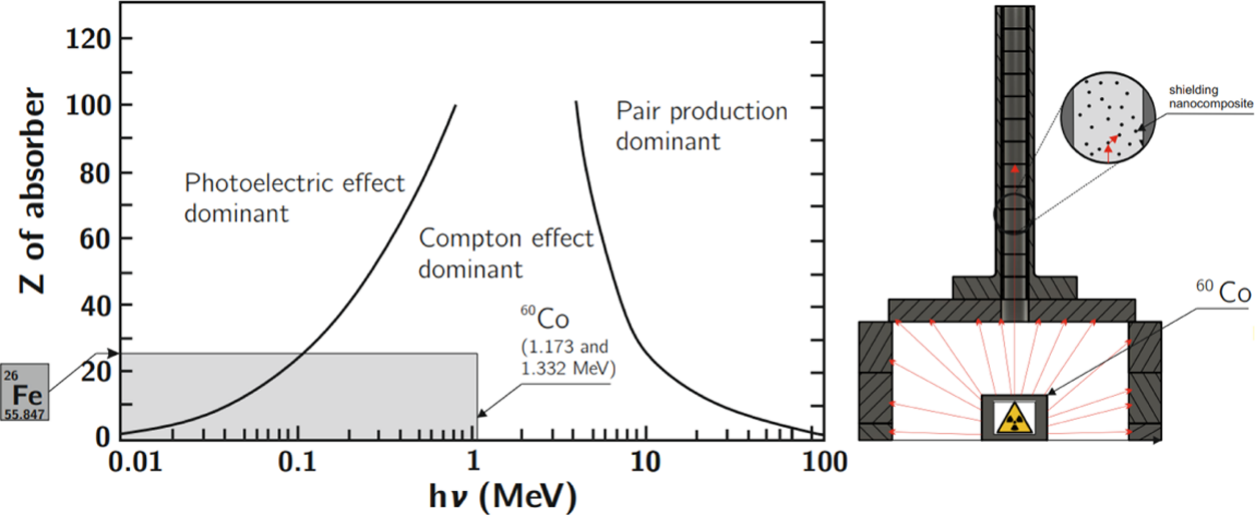
Dependence of the occurrence of individual phenomena on the value of the atomic number. The selected field is narrowed down to the phenomena that concern the examined nano- and microcomposites.

γ Radiation can interact with both atomic nuclei and electrons (strongly bound in atoms or valence), leading to scattering (elastic or inelastic) or direct absorption. The photoelectric effect (prevails at low energy), the Compton effect (predominates in the intermediate energies), and the formation of an electron–positron pair (occurs at high radiation energy) are among the most important processes involving the quenching of γ-radiation.^41^ Analyzing the shielding mechanism in our nano- and microcomposites, the Compton effect is dominant considering γ radiation and the photoelectric effect with the Compton effect in X-rays. The probability of electromagnetic radiation interactions, depending on the energy value and the atomic number of the shielding material, is shown in Figure 17 (left), with a schematic diagram of a chamber for γ-ray studies, Figure 17 (right).

**Figure 18:**
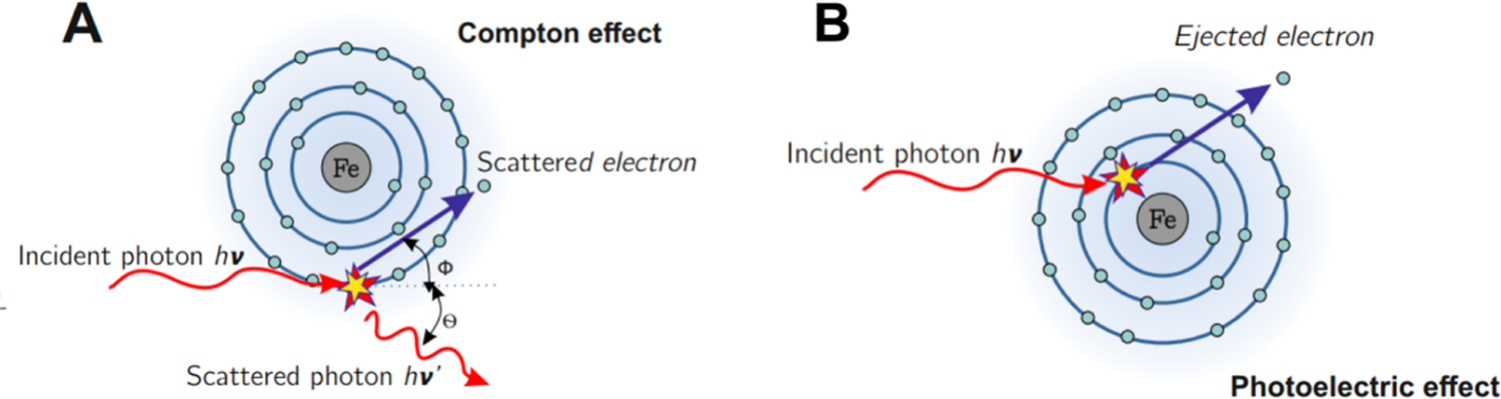
Schematic representation of the interaction of electromagnetic radiation: Compton effect (A) and photoelectric absorption (B).

Considering the γ-radiation, the Compton effect occurs, which is responsible for the shielding ability of the manufactured nanocomposites. The incident γ radiation quantum is scattered on the electron in the iron atoms. As a result of the interaction, part of the energy is transferred to the electron, which is also being scattered, while the γ-radiation quantum changes the direction of its propagation and momentum in accordance with the principle of conservation of energy and momentum. A simplified drawing based on the Bohr model of the atom is shown in Figure 18.

On the other hand, in the lower energy range, two mechanisms take place: the Compton effect and the photoelectric effect. The photoelectric effect occurs when the energy of the incident γ radiation exceeds the binding energy of electrons in a given shell. The incident quantum of radiation interacts with the atom, causing it to become excited, and the excess energy is released by ejecting one of the electrons. The ejected electron is called a photoelectron. A graphical representation of the photoelectric effect, also based on the simplified Bohr model of the atom, is shown in Figure 18.

## Conclusions

We have designed and elaborated a convenient manufacturing process for paraffin-based nano- and microcomposites containing iron particles as shielding materials in numerous scientific, industrial, and medical applications. The manufactured samples were stable and, more importantly, easily formable at room temperature, which drastically distinguishes them from the previously proposed materials. It is also worth mentioning the ease and simplicity of recovering individual components from nano- and microcomposites for reuse. Heating the composite to a temperature above the paraffin melting point will convert the paraffin from a solid to a liquid, which, at the same time, will enable the sedimentation of the iron nanoparticles. As a less toxic^42,43^ and eco-friendly shield, prospective nano- and microcomposites represent a reliable attempt to replace the most abundant lead-based shields in daily life. The obtained data showed that the size of the used iron particles in the composite had a negligible effect on the shielding properties, and the nanocomposites protect against γ radiation similarly to microcomposites, which undoubtedly decreases the production costs of such materials.

The comprehensively developed procedure is inexpensive, reproducible, and scalable up to the industrial scale. The most important benefits of using the presented material include:

(a) ease of forming any shape of the cover: the shapes of the composite can be changed with bare hands and manufactured to any specific ones, which is particularly important, e.g., in the medical sector in the individual protection of patients and staff,
(b) straightforward recycling and eco-friendliness: the cover can be processed/combined with materials in the container with bare hands, and, despite the possibility of commercial purchase, iron nanoparticles can be obtained from waste or synthesized using green chemistry,^44−46^
(c) independence from the specialized apparatus: the technological process is relatively simple and broadly available, not requiring a large amount of energy.

Above all, studies of novel nano- and microcomposites dedicated to radiological protection are a promising scientific direction. The formability of our nano- and microcomposites can be potentially used as personalized shields, e.g., in the form of gloves or aprons for staff and patients, e.g., thyroid covers in the case of mammography or gonads while taking X-ray images of breast, etc. Likewise, they can be used as shields for employees in research and development centers or in industry as well as an auxiliary element characterized by an unusual geometry, e.g., in the shielding structure of radioactive sources (up to 1 MeV emitted energy).

Future work should focus on the determination of linear attenuation coefficients for a broader scope of particles. The efforts should also be aimed at the design, manufacturing, and examination of the composites containing other nano- and microparticles of high mass number elements and their compounds displaying minimal toxicity.

## Supporting Information Available

The Supporting Information includes: detailed picture of manufacturing procedure of producing microcomposites, XRD analysis, NMR spectra, TGA curves, EDX results, γ and X-ray attenuation plots of nano- and microcomposites together with tabulated data (PDF)
Movie showing the possibility shape reforming of nano- and microcomposites (MP4)

## References

[ref1] World Health Organization. International Basic Safety Standards for Protection against Ionizing Radiation and for the Safety of Radiation Sources. IAEA Safety Series No. 115; International Labour Organization (ILO) 1994 19-27.

[ref2] Faw R. E.; Shultis K. Radiation Shielding; American Nuclear Society, Inc.: La Grange Park, IL, 2000 60526.

[ref3] Hsissou R.; Seghiri R.; Benzekri Z.; Hilali M.; Rafik M.; Elharfi A. Polymer Composite Materials: A Comprehensive Review. Compos. Struct. 2021, 262, 113640 10.1016/j.compstruct.2021.113640.

[ref4] Mutombo P.; Hackerman N. Potential Decay Behavior of Iron in Dilute Nitric Acid. J. Solid State Electrochem. 1997, 1 (3), 194–198. 10.1007/s100080050048.

[ref5] Banaś J. Passivity of Iron and Nickel in a CH_3_OH-H_2_O-H_2_SO_4_ System. Electrochim. Acta 1987, 32 (6), 871–875. 10.1016/0013-4686(87)87076-7.

[ref6] Oka Y.; An S.; Kasai S.; Miyasaka S.; Koyama K. Neutron and Gamma-Ray Penetrations in Thick Iron. Nucl. Sci. Eng. 1980, 73 (3), 259–273. 10.13182/NSE80-A19850.

[ref7] Verhiest K.; Almazouzi A.; Wispelaere N. D.; Petrov R.; Claessens S. Development of Oxides Dispersion Strengthened Steels for High Temperature Nuclear Reactor Applications. J. Nucl. Mater. 2009, 385 (2), 308–311. 10.1016/j.jnucmat.2008.12.006.

[ref8] Hishinuma A.; Isozaki S.; Takaki S.; Abiko K. Attractive Characteristics of High-Chromium Iron-Based Alloys for Nuclear Reactor Application. Phys. Status Solidi A 1997, 160 (2), 431–440. 10.1002/1521-396X(199704)160:2<431::AID-PSSA431>3.0.CO;2-B.

[ref9] Sathish K. V.; Manjunatha H. C.; Vidya Y. S.; Sridhar K. N.; Seenappa L.; Reddy B. C.; Raj S. A. C.; Gupta P. S. D. X-Rays/Gamma Rays Radiation Shielding Properties of Barium–Nickel–Iron Oxide Nanocomposite Synthesized via Low Temperature Solution Combustion Method. Radiat. Phys. Chem. 2022, 194, 110053 10.1016/j.radphyschem.2022.110053.

[ref10] El-Khatib A. M.; Abbas M. I.; Elzaher M. A.; Anas M.; El Moniem M. S. A.; Montasar M.; Ellithy E.; Alabsy M. T. A New Environmentally Friendly Mortar from Cement, Waste Marble and Nano Iron Slag as Radiation Shielding. Materials 2023, 16 (7), 2541 10.3390/ma16072541. 37048835 PMC10095434

[ref11] AL-Rajhi M. A.; Idriss H.; Alaamer A.-A. S.; El-Khayatt A. M. Gamma/Neutron Radiation Shielding, Structural and Physical Characteristics of Iron Slag Nanopowder. Appl. Radiat. Isot. 2021, 170, 109606 10.1016/j.apradiso.2021.109606. 33571735

[ref12] Reda A. M.; Azab A. A.; Turky G. M. Gamma-Ray Shielding, Electrical, and Magnetic Properties of α-Fe_2_O_3_/Al/HDPE Nanocomposites. Phys. Scr. 2022, 97 (9), 095303 10.1088/1402-4896/ac8458.

[ref13] Badawy S. M.; El-Latif A. A. A. Synthesis and Characterizations of Magnetite Nanocomposite Films for Radiation Shielding. Polym. Compos. 2017, 38 (5), 974–980. 10.1002/pc.23660.

[ref14] Rainwater J.; Havens W. W. Neutron Beam Spectrometer Studies of Boron, Cadmium, and the Energy Distribution from Paraffin. Phys. Rev. 1946, 70 (3–4), 136–153. 10.1103/PhysRev.70.136.

[ref15] Turner W. R.; Brown D. S.; Harrison D. V. Properties of Paraffin Waxes. Ind. Eng. Chem. 1955, 47 (6), 1219–1226. 10.1021/ie50546a049.

[ref16] Soliman F. S. Paraffin: An Overview. In Petrochemical Engineering; IntechOpen, 2020.

[ref17] Wei H.; Lou L.; Yang Z.; He R.; Fan J.; Zhang K.; Yang W. Multifunctional Composites Silicone Rubber/Paraffin@ Lead Tungstate with Different Core/Shell Ratio for Thermal Regulation and Gamma Shielding. J. Energy Storage 2021, 36, 102363 10.1016/j.est.2021.102363.

[ref18] Lou L.; Jiang Z.; Zhang Q.; Liu D.; Zhou Y.; Zhang K.; He R.; Fan J.; Yan H.; Yang W. Phase Change Microcapsules with Lead Tungstate Shell for Gamma Radiation Shielding and Thermal Energy Storage. Int. J. Energy Res. 2019, 43 (14), 8398–8409. 10.1002/er.4838.

[ref19] Bąk I.; Cheba K. Green Energy: Meta-Analysis of the Research Results, Green Energy and Technology; Springer, 2023.

[ref20] Ikram R.; Jan B. M.; Nagy P. B.; Szabo T. Recycling Waste Sources into Nanocomposites of Graphene Materials: Overview from an Energy-Focused Perspective. Nanotechnol. Rev. 2023, 12 (1), 20220512 10.1515/ntrev-2022-0512.

[ref21] Lee C.-W.; Lin C.-H.; Wang L.-Y.; Lee Y.-H. Developing Sustainable and Recyclable High-Efficiency Electromagnetic Interference Shielding Nanocomposite Foams from the Upcycling of Recycled Poly (Ethylene Terephthalate). Chem. Eng. J. 2023, 468, 143447 10.1016/j.cej.2023.143447.

[ref22] Shaikh W. A.; Chakraborty S.; Islam R. U.; Ghfar A. A.; Naushad M.; Bundschuh J.; Maity J. P.; Mondal N. K. Fabrication of Biochar-Based Hybrid Ag Nanocomposite from Algal Biomass Waste for Toxic Dye-Laden Wastewater Treatment. Chemosphere 2022, 289, 133243 10.1016/j.chemosphere.2021.133243. 34896417

[ref23] Barasarathi J.; Abdullah P. S.; Uche E. C. Application of Magnetic Carbon Nanocomposite from Agro-Waste for the Removal of Pollutants from Water and Wastewater. Chemosphere 2022, 305, 135384 10.1016/j.chemosphere.2022.135384. 35724716

[ref24] El-Sharkawy R. M.; Abdou F. S.; Gizawy M.; Allam E. A.; Mahmoud M. E. Bismuth Oxide Nanoparticles (Bi_2_O_3_ NPs) Embedded into Recycled-Poly (Vinyl Chloride) Plastic Sheets as a Promising Shielding Material for Gamma Radiation. Radiat. Phys. Chem. 2023, 208, 110838 10.1016/j.radphyschem.2023.110838.

[ref25] Mahmoud M. E.; El-Khatib A. M.; Halbas A. M.; El-Sharkawy R. M. Ceramic Tiles Doped with Lead Oxide Nanoparticles: Their Fabrication, Physical, Mechanical Characteristics and γ-Ray Shielding Performance. Radiat. Phys. Chem. 2021, 189, 109780 10.1016/j.radphyschem.2021.109780.

[ref26] El-Khatib A. M.; Abbas Y. M.; Badawi M. S.; Hagag O. M.; Alabsy M. T. Gamma Radiation Shielding Properties of Recycled Polyvinyl Chloride Composites Reinforced with Micro/Nano-Structured PbO and CuO Particles. Phys. Scr. 2021, 96 (96), 125316 10.1088/1402-4896/ac35c3.

[ref27] Prabhu S.; Buddy S. G.; Gudennavar S. B. X-Ray and γ-Ray Shielding Efficiency of Polymer Composites: Choice of Fillers, Effect of Loading and Filler Size, Photon Energy and Multifunctionality. Polym. Rev. 2023, 63 (1), 246–288. 10.1080/15583724.2022.2067867.

[ref28] Urlaub J.; Norwig J.; Schollmayer C.; Holzgrabe U. ^1^H NMR Analytical Characterization of Mineral Oil Hydrocarbons (PARAFFINS) for Pharmaceutical Use. J. Pharm. Biomed. Anal. 2019, 169, 41–48. 10.1016/j.jpba.2019.01.036. 30831451

[ref29] Wang L.; Lu X.; Han C.; Lu R.; Yang S.; Song X. Electrospun Hollow Cage-like α - Fe_2_O_3_ Microspheres: Synthesis, Formation Mechanism, and Morphology-Preserved Conversion to Fe Nanostructures. CrystEngComm 2014, 16, 10618–10623. 10.1039/C4CE01485E.

[ref30] Mazur J.; Fanconi B. Raman Spectra of N-Alkanes. I. Raman Intensities of Longitudinal Acoustic Modes. J. Chem. Phys. 1979, 71 (12), 5069–5081. 10.1063/1.438280.

[ref31] Puerto M. A.; Balzaretti N. M. Raman and Infrared Vibrational Modes of Tricosane Paraffin under High Pressure. Vib. Spectrosc. 2014, 75, 93–100. 10.1016/j.vibspec.2014.10.004.

[ref32] Alpen E. L. Radiation Biophysics; Academic press, 1997.

[ref33] Akkurt I.; Kilincarslan S.; Basyigit C. The Photon Attenuation Coefficients of Barite, Marble and Limra. Ann. Nucl. Energy 2004, 31 (5), 577–582. 10.1016/j.anucene.2003.07.002.

[ref34] Belgin E. E.; Aycik G. A.; Kalemtas A.; Pelit A.; Dilek D. A.; Kavak M. T. Preparation and Characterization of a Novel Ionizing Electromagnetic Radiation Shielding Material: Hematite Filled Polyester Based Composites. Radiat. Phys. Chem. 2015, 115, 43–48. 10.1016/j.radphyschem.2015.06.008.

[ref35] Aygün B. Neutron and Gamma Radiation Shielding Properties of High-Temperature-Resistant Heavy Concretes Including Chromite and Wolframite. J. Radiat. Res. Appl. Sci. 2019, 12 (1), 352–359. 10.1080/16878507.2019.1672312.

[ref36] Florez R.; Colorado H. A.; Alajo A.; Giraldo C. H. The Material Characterization and Gamma Attenuation Properties of Portland Cement-Fe_3_O_4_ Composites for Potential Dry Cask Applications. Prog. Nucl. Energy 2019, 111, 65–73. 10.1016/j.pnucene.2018.10.022.

[ref37] Qadr H. M. Calculation of gamma-ray attenuation parameters for aluminium, iron, zirconium and tungsten. Probl. At. Sci. Technol., Ser.: Plasma Phys. 2020, 43 (2), 25–30. 10.21517/0202-3822-2020-43-2-25-30.

[ref38] Gouda M. M.; El-Khatib A. M.; Abbas M. I.; Al-Balawi S. M.; Alabsy M. T. Gamma Attenuation Features of White Cement Mortars Reinforced by Micro/Nano Bi_2_O_3_ Particles. Materials 2023, 16 (4), 1580 10.3390/ma16041580. 36837210 PMC9966324

[ref39] Mahmoud M. E.; El-Khatib A. M.; Badawi M. S.; Rashad A. R.; El-Sharkawy R. M.; Thabet A. A. Recycled High-Density Polyethylene Plastics Added with Lead Oxide Nanoparticles as Sustainable Radiation Shielding Materials. J. Cleaner Prod. 2018, 176, 276–287. 10.1016/j.jclepro.2017.12.100.

[ref40] Cheewasukhanont W.; Limkitjaroenporn P.; Kothan S.; Kedkaew C.; Kaewkhao J. The Effect of Particle Size on Radiation Shielding Properties for Bismuth Borosilicate Glass. Radiat. Phys. Chem. 2020, 172, 108791 10.1016/j.radphyschem.2020.108791.

[ref41] Malekie S.; Hajiloo N. Comparative Study of Micro and Nano Size WO_3_/E44 Epoxy Composite as Gamma Radiation Shielding Using MCNP and Experiment. Chin. Phys. Lett. 2017, 34 (10), 108102 10.1088/0256-307X/34/10/108102.

[ref42] Kim S.-G.; Kang J. W.; Boo J. H.; Jin D. U.; Choi S. J.; Song G. G.; Jung J. H. Effectiveness of Paraffin Bath Therapy for the Symptoms and Function of Hand Diseases: A Systematic Review and Meta-Analysis of Randomized Controlled Trials. J. Hand Ther. 2023, 36 (3), 706–712. 10.1016/j.jht.2022.10.005. 36914488

[ref43] Yan W.; Liu L.; Yang T.; Yang X. Traditional Chinese Medicine Paraffin Therapy: An Evidence-Based Overview from a Modern Medicine Perspective. Chin. Med. 2022, 17 (1), 106 10.1186/s13020-022-00662-z. 36104753 PMC9476693

[ref44] Kharissova O. V.; Dias H. V. R.; Kharisov B. I.; Pérez B. O.; Pérez V. M. J. The Greener Synthesis of Nanoparticles. Trends Biotechnol. 2013, 31 (4), 240–248. 10.1016/j.tibtech.2013.01.003. 23434153

[ref45] Borth K. W.; Galdino C. W.; Teixeira V. D. C.; Anaissi F. J. Iron Oxide Nanoparticles Obtained from Steel Waste Recycling as a Green Alternative for Congo Red Dye Fast Adsorption. Appl. Surf. Sci. 2021, 546, 149126 10.1016/j.apsusc.2021.149126.

[ref46] Saif S.; Tahir A.; Chen Y. Green Synthesis of Iron Nanoparticles and Their Environmental Applications and Implications. Nanomaterials 2016, 6 (11), 209 10.3390/nano6110209. 28335338 PMC5245755

