# Supplementary material for: Shape-Controlled Iron–Paraffin Composites as γ- and X-ray Shielding Materials Formable by Warmth-of-Hands-Derived Plasticity
Source: ACS Appl Eng Mater. 2023 Dec 1;1(12):3237–53. doi: 10.1021/acsaenm.3c00557 (PMC10749452; doi:10.1021/acsaenm.3c00557)
Supplement: Supplementary file 1 — em3c00557_si_001.pdf [file em3c00557_si_001.pdf]

Supporting Information

Shape-controlled Iron-paraffin Composites as gamma- and X-ray Shielding Materials Formable by Warmth-of-hands-derived Plasticity

Jolanta Sobczak[a], Adrian Truszkiewicz[b], Emil Korczeniewski[c], Aleksandra Cyganiuk[c], Artur P. Terzyk[c*], Anna Kolanowska[d,e], Rafał G. Jędrysiak[d,f], Sławomir Boncel[d,f*], Gaweł Żyła[g*]

[a] *Doctoral School of the Rzeszów University of Technology, Rzeszów University of Technology, 35-959 Rzeszów, Poland*

[b] *Department of Photomedicine and Physical Chemistry, Medical College of University of Rzeszow, University of Rzeszow, Warzywna 1A Street, 35-310 Rzeszów, Poland*

[c] *Faculty of Chemistry, Physicochemistry of Carbon Materials Research Group, Nicolaus Copernicus University in Torun, Gagarin Street 7, 87-100 Torun, Poland*

[d] *Department of Organic Chemistry, Bioorganic Chemistry and Biotechnology, Silesian University of Technology, 44-100 Gliwice, Poland*

[e] *Biotechnology Centre, Silesian University of Technology, 44-100 Gliwice, Poland*

[f] *Centre for Organic and Nanohybrid Electronics, Silesian University of Technology, 44-100 Gliwice, Poland*

[g] *Department of Physics and Medical Engineering, Rzeszow University of Technology, 35-959 Rzeszow, Poland*

* Corresponding authors:

E-mail addresses: aterzyk@chem.umk.pl; slawomir.boncel@polsl.pl; gzyla@prz.edu.pl.

Manufacturing procedure

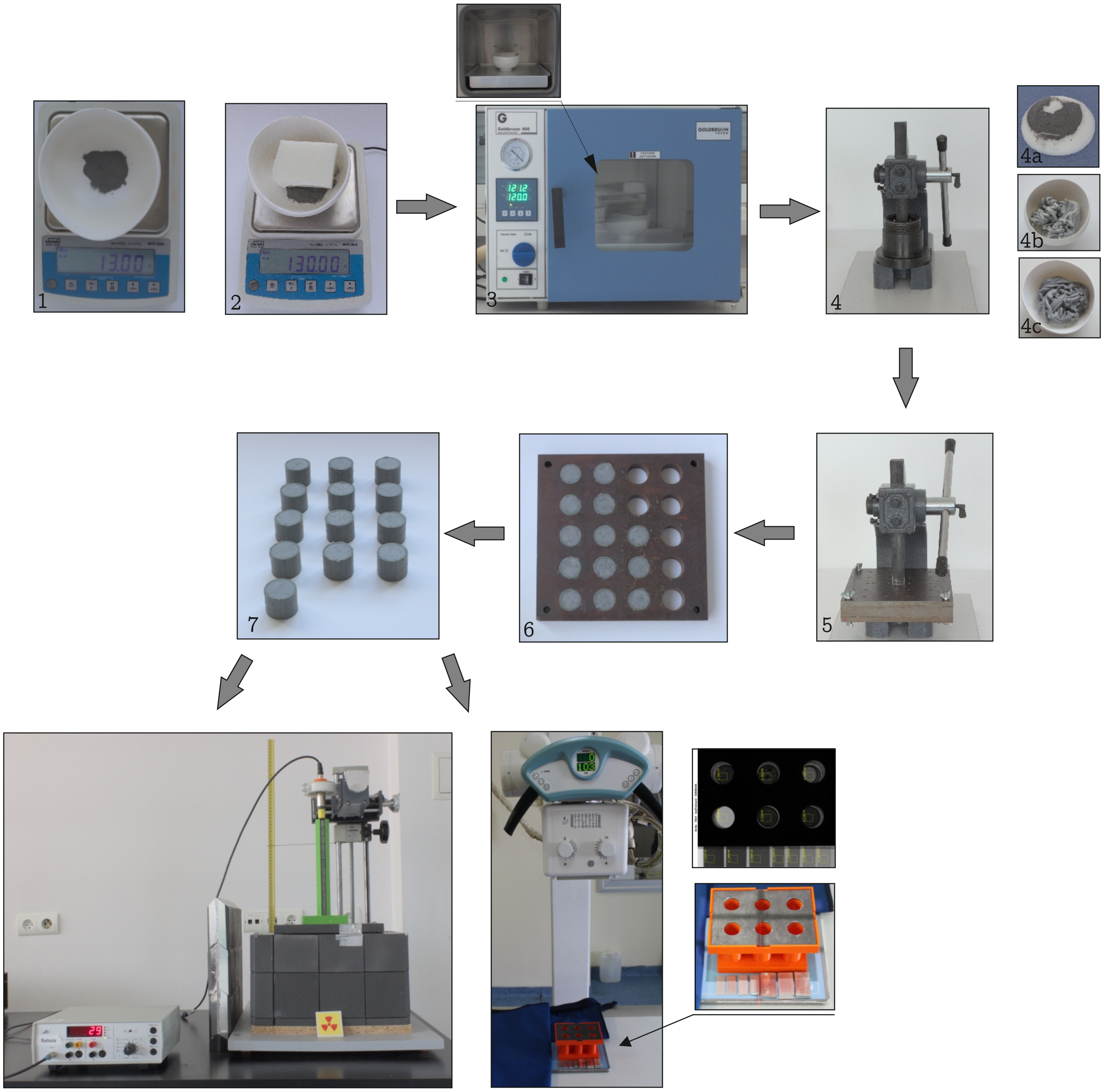

Figure S1: The process of producing microcomposites on the example of a composite with 10 wt.% addition of iron microparticles (size 63μm): 1. weighing the right amount of iron micropowder, 2. addition of the paraffin, 3. placing the sample in a vacuum dryer in order to remove air bubbles from the paraffin 4. cold mixing with a hand press, with a view of the sample before (4a), during (4b) and after mixing (4c), 5. pressing a system of three metal molds with placed sample rings. The Pic. 6 presents a top view of the placed rings, and 7. achieved samples, which have been experimentally measured for the absorption of gamma (left bottom picture) and X radiation (right bottom picture).

XRD analysis

Table S1: Name and formula.

Reference code:	00-006-0696
Mineral name:	Iron, syn
Common name:	ferrite
PDF index name:	Iron
Empirical formula:	Fe
Chemical formula:	Fe

Table S2: Crystallographic parameters.

Crystal system:	Cubic
Space group:	Im-3m
Space group number:	229
a (Å):	2.8664
b (Å):	2.8664
c (Å):	2.8664
Alpha (°):	90.0000
Beta (°):	90.0000
Gamma (°):	90.0000
Calculated density (g·cm^{-3}):	7.87
Volume of cell (10^6 pm^3):	23.55
Z:	2.00
RIR:	—

Table S3: Subfiles and Quality.

Subfiles:	Inorganic
	Mineral
	Alloy, metal or intermetalic
	Common Phase
	Educational pattern
	Forensic
	NBS pattern
Quality:	Star (S)

Table S4: Comments.

Color:	Gray, light gray metallic
General comments:	Total impurities of sample <0.0013% each metals and non-metals. g-Fe (fcc)=(1390 °C) δ-Fe (bcc). Opaque mineral optical data on specimen from Meteorite: RR_2R_e= 57.7, Disp.=16, VHN=158 (mean at 100, 200, 300), Color values=.311, .316, 57.9, Ref.: IMA Commisssion on Ore Microscopy QDF.
Sample preparation:	The iron used was an exceptionally pure rolled sheet prepared at the NBS, Gaithersburg, Maryland, USA., [Moore, G., *J. Met.*, 5 1443 (1953)]. It was annealed in an H_2 atmosphere for 3 days at 1100 °C and slowly cooled in a He atmosphere.
Additional pattern:	See ICSD 64795 (PDF 85-1410).
Temperature:	Pattern taken at 25 °C.

Table S5: References.

Primary reference:	Swanson et al., Natl. Bur. Stand. (U.S.), Circ. 539, IV, 3, (1955)

Table S6: Peak list.

No.	h	k	l	d [A]	2Theta[deg]	I [%]
1	1	1	0	2.02680	44.674	100.0
2	2	0	0	1.43320	65.023	20.0
3	2	1	1	1.17020	82.335	30.0
4	2	2	0	1.01340	98.949	10.0
5	3	1	0	0.90640	116.390	12.0
6	2	2	2	0.82750	137.144	6.0

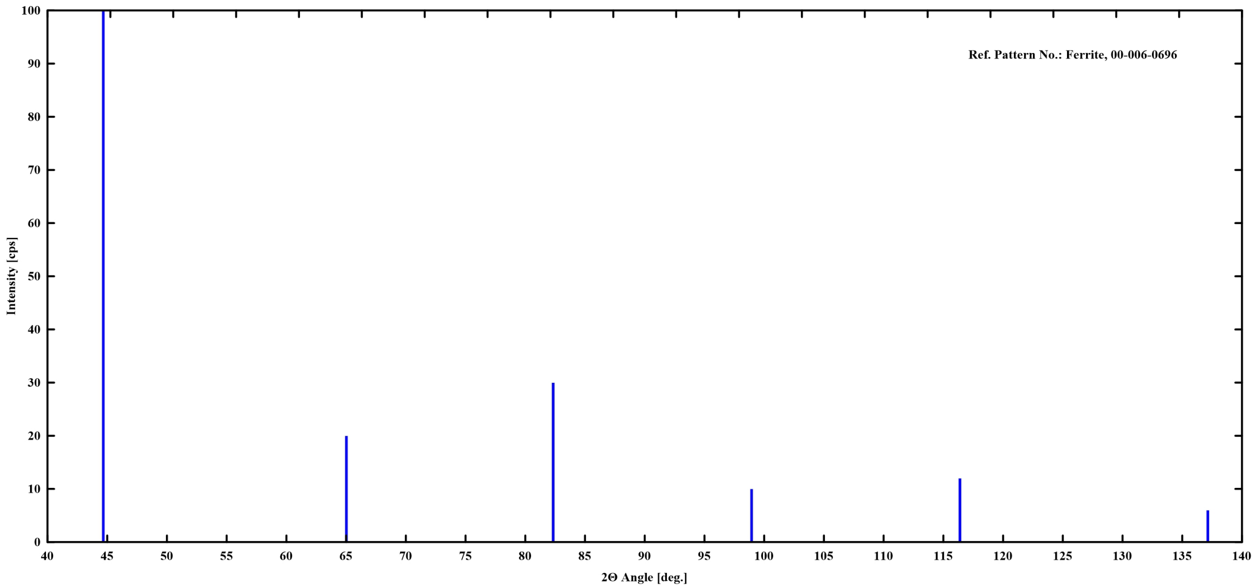

Figure S2: Stick Pattern.

Table S7: Name and formula.

Reference code:	00-013-0675
Mineral name:	Unnamed mineral [NR]
PDF index name:	Paraffin
Empirical formula:	CH_2
Chemical formula:	$(CH_2)n$

Table S8: Crystallographic parameters.

Crystal system:	Monoclinic
Space group:	P21/a
Space group number:	14
a (Å):	7.5900
b (Å):	4.9800
c (Å):	33.8000
Alpha (°):	90.0000
Beta (°):	92.2500
Gamma (°):	90.0000
Calculated density (g·cm^{-3}):	0.78
Measured density (g·cm^{-3}):	0.87
Volume of cell (10^6 pm^3):	1276.59
Z:	2.00
RIR:	—

Table S9: Subfiles and Quality.

Subfiles:	Organic
	Mineral
	Polymer
Quality:	Blank (B)

Table S10: Comments.

Color:	Colorless, light yellow.
General comments:	b reflections are omitted.
Sample source:	A natural crystalline paraffin wax from Lower Tunguska, Siberia, occurring in quartz nodules in polymetallic ore, which are found in lava deposits of tuffaceous strata.
Analysis:	Chemical analysis (wt.%): C 85.43, H 14.66.
Optical data:	A= 1.504, B= 1.504, Q=1.533
Melting point:	49-52°C

Table S11: References.

Primary reference:	Mikheev, V., X-ray Determination of Minerals, 819, (1957)
Unit cell:	Strunz et al. (refined), Neues Jahrb. Mineral., Monatsh., 19, (1965)
Powder data:	Skropyshev, A., Dopov. Akad. Nauk Ukr. RSR, 88, 717, (1953)

Table S12: Peak list.

No.	h	k	l	d [A]	2Theta[deg]	I [%]
1				9.36000	9.441	10.0
2	1	1	0	4.18000	21.239	100.0
3	-2	0	2	3.74000	23.772	90.0
4	-2	0	5	3.35000	26.587	10.0
5	-2	1	0	3.02000	29.555	50.0
6	2	1	6	2.63000	34.062	10.0
7	2	1	7	2.52000	35.598	70.0
8	0	2	2	2.46000	36.496	20.0
9	2	1	8	2.42000	37.121	40.0
10	0	0	15	2.25000	40.041	80.0
11	0	2	8	2.15000	41.989	30.0
12	2	1	11	2.12000	42.612	60.0
13	-2	2	1	2.08000	43.473	30.0
14	-3	1	8	2.02000	44.833	30.0
15	-2	1	14	1.91400	47.464	50.0
16	-4	0	4	1.86600	48.763	30.0
17	-4	1	4	1.75100	52.198	60.0
18	-4	0	9	1.71800	53.278	10.0
19	0	3	1	1.65800	55.368	50.0
20	1	1	19	1.62300	56.669	30.0
21	2	1	18	1.57100	58.724	10.0
22	3	1	16	1.51500	61.121	40.0
23	2	0	21	1.46000	63.687	20.0
24	4	2	7	1.42600	65.392	20.0
25	-2	1	22	1.38600	67.528	30.0
26	0	0	26	1.29900	72.740	40.0
27	6	0	4	1.24300	76.590	40.0
28	-3	3	14	1.21400	78.768	40.0
29				1.11600	87.297	40.0
30				1.10200	88.694	20.0
31				1.02600	97.316	30.0

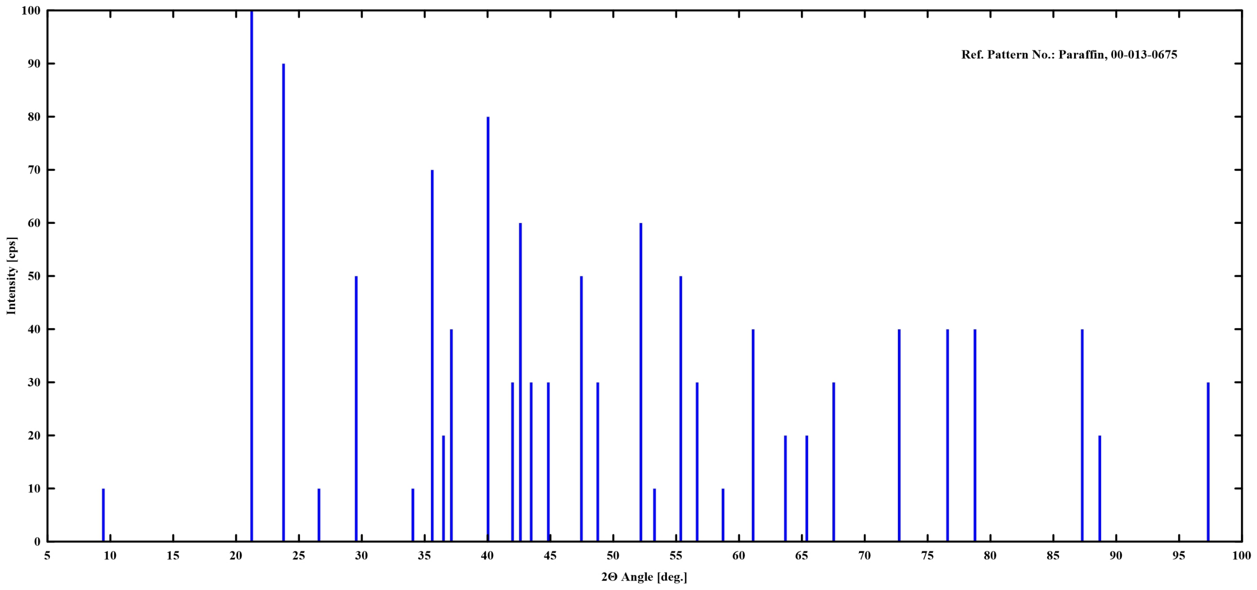

Figure S3: Stick Pattern.

NMR spectra

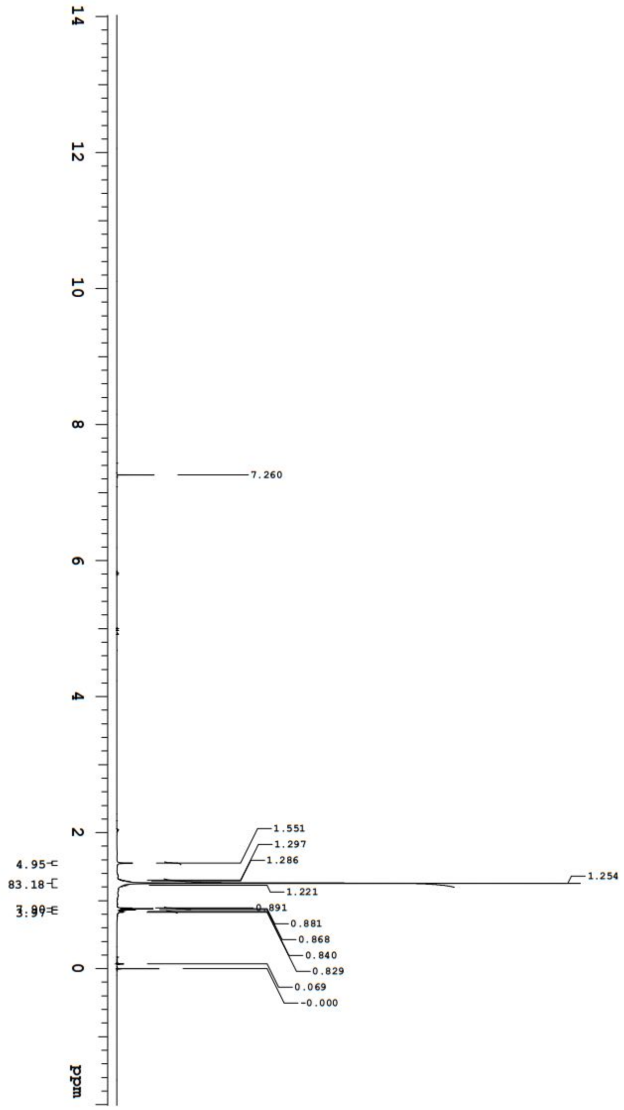

Figure S4: ^{1}H NMR spectrum of paraffin in the 14 - 0 ppm signal range.

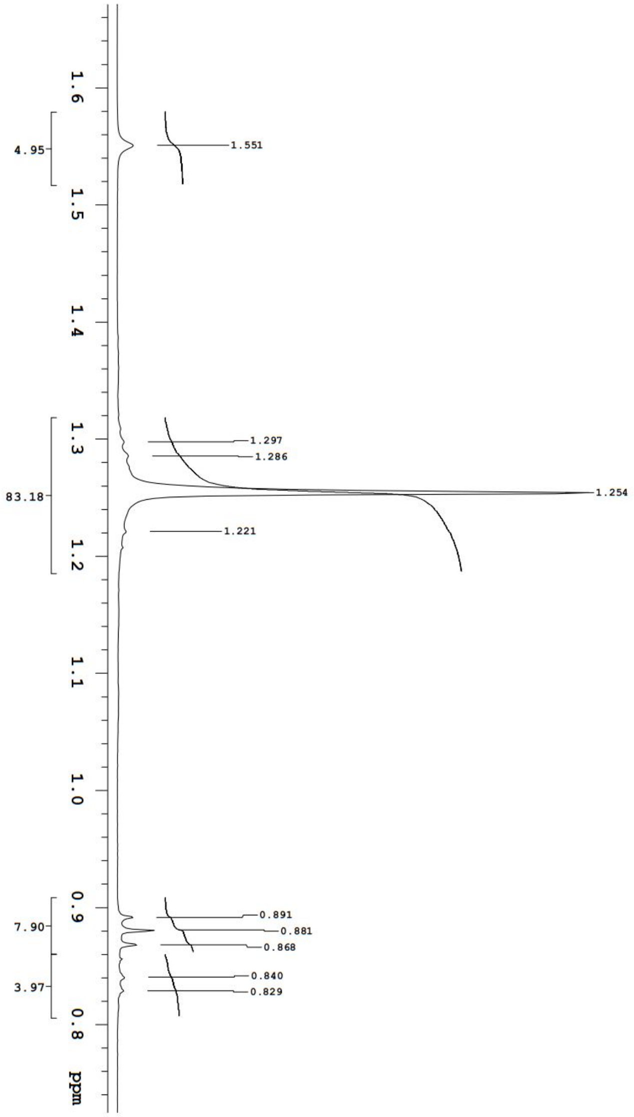

Figure S5: ^1H NMR spectrum of paraffin in the 1.6 - 0.8 ppm signal range.

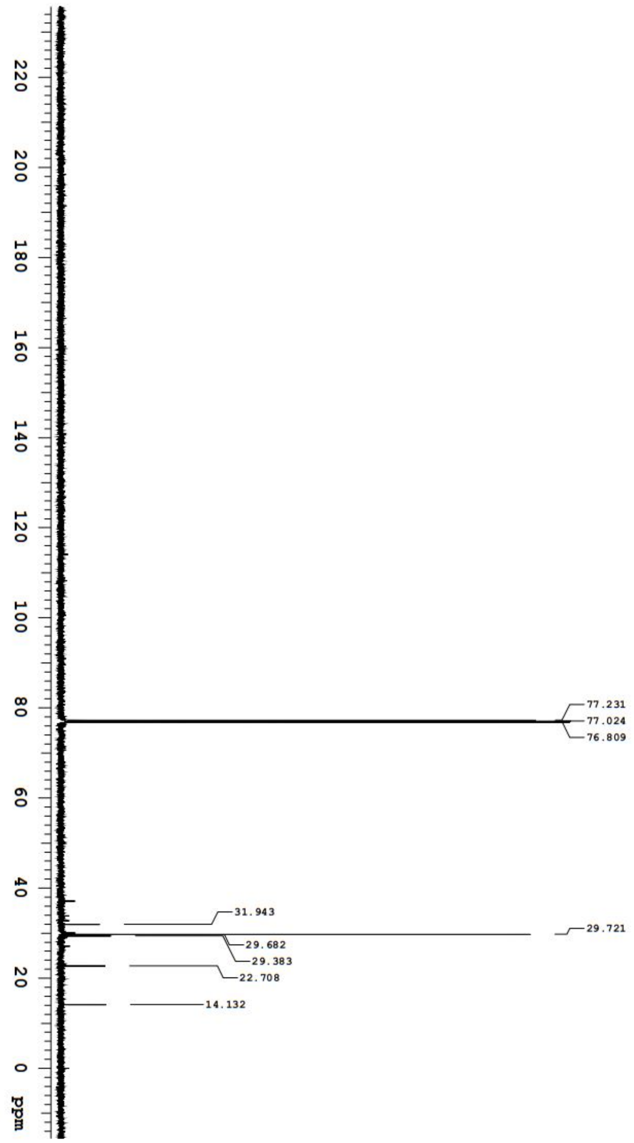

Figure S6: ^{13}C NMR spectrum of paraffin in the 220 - 0 ppm signal range.

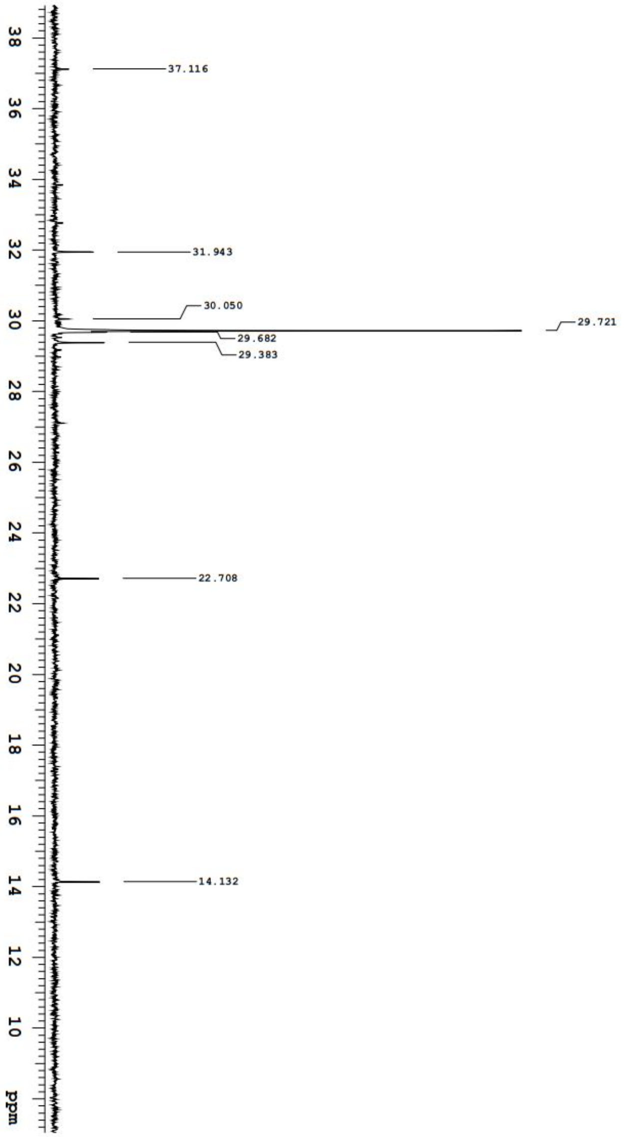

Figure S7: ^{13}C NMR spectrum of paraffin in the 38 - 10 ppm signal range.

Thermal analysis results

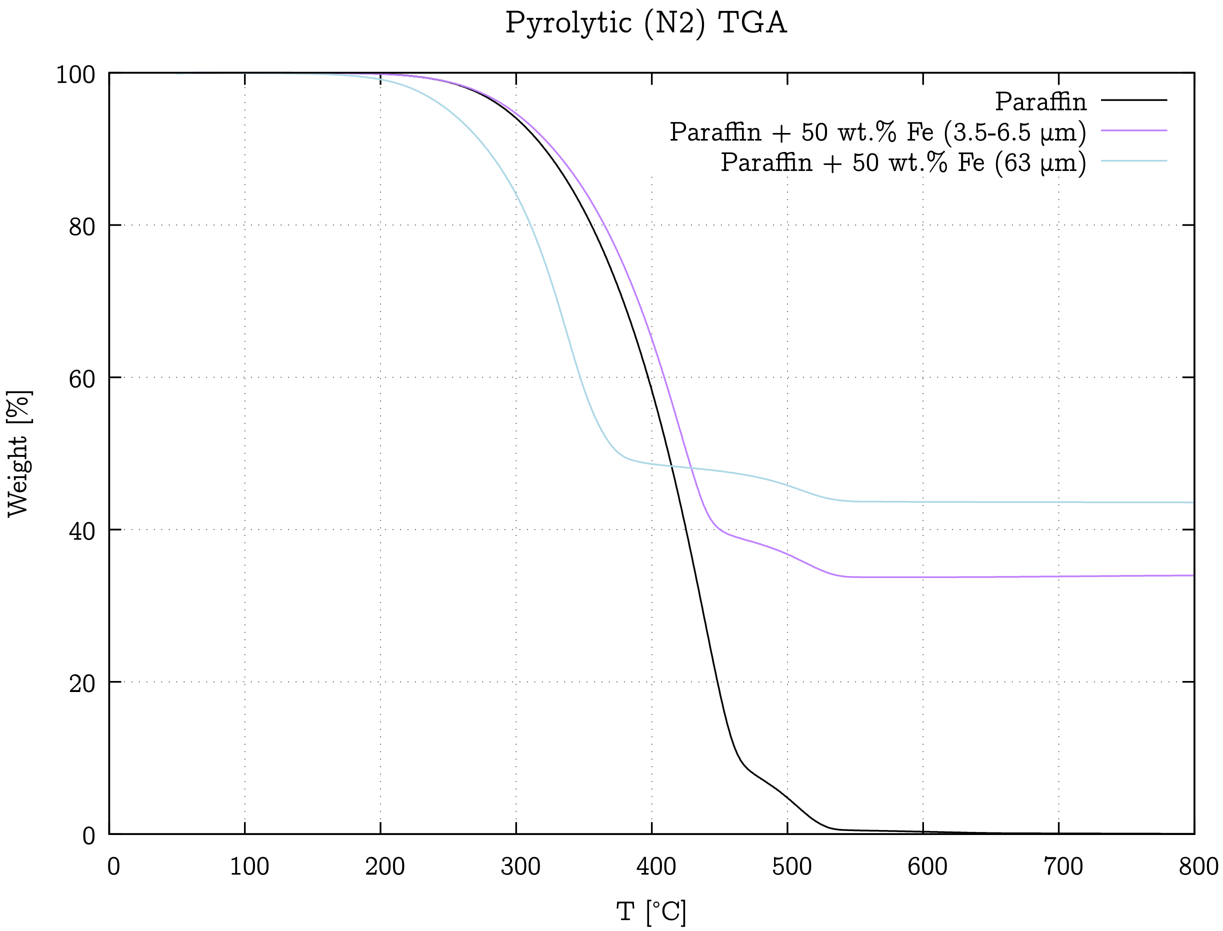

Figure S8: Exemplary TGA curve for paraffin and the Paraffin + Fe composite.

EDX results

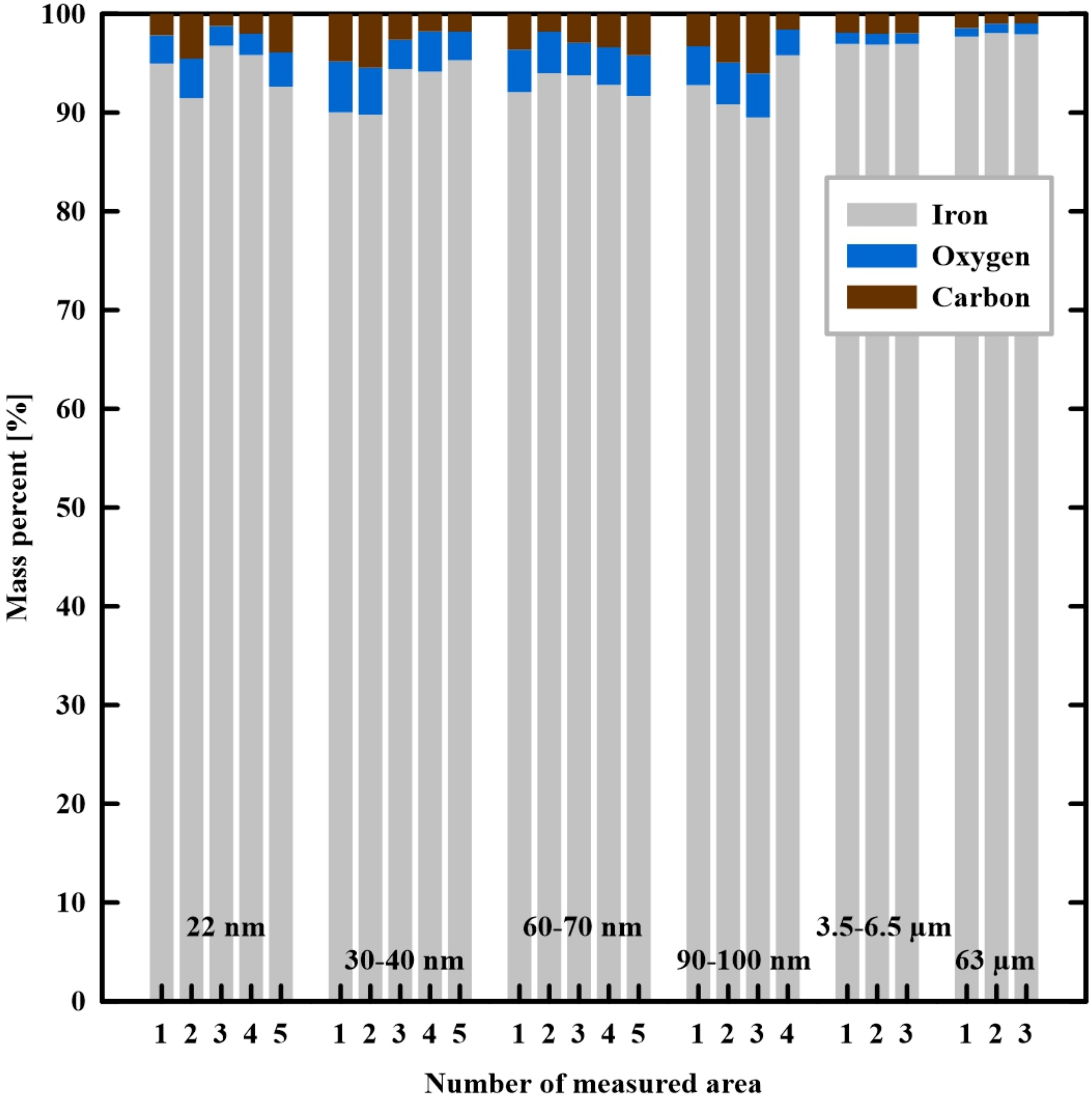

Figure S9: EDX results for studied samples.

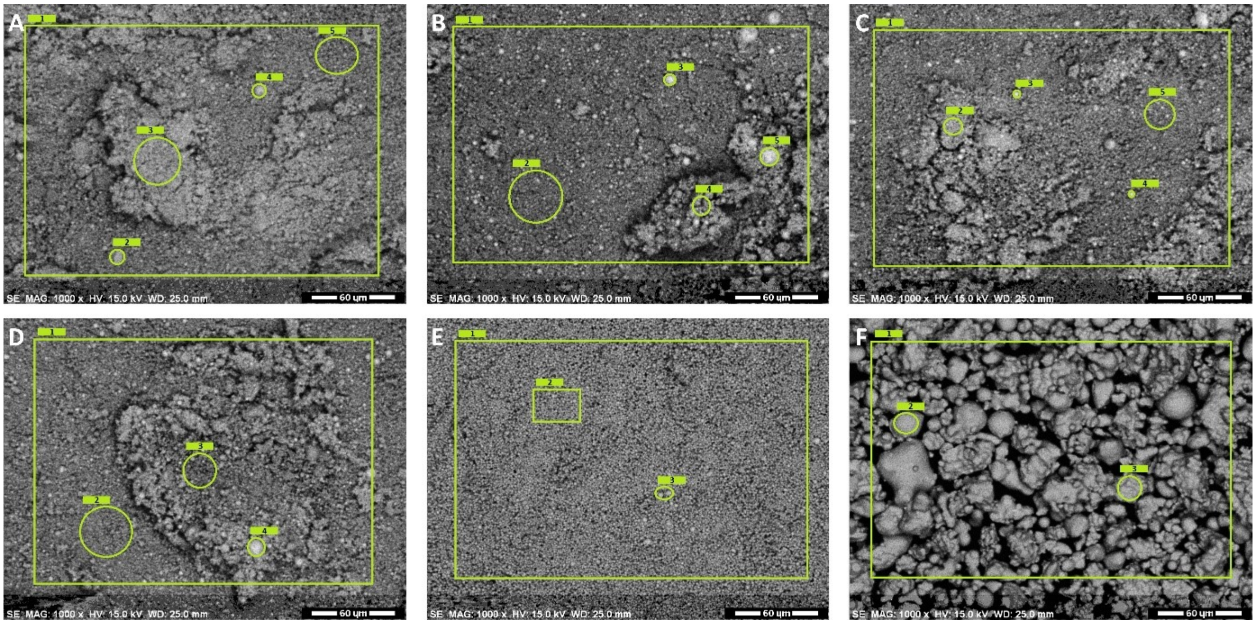

Figure S10: SEM pictures with marked areas for EDX measurements for studied samples.

Shielding properties - gamma-ray

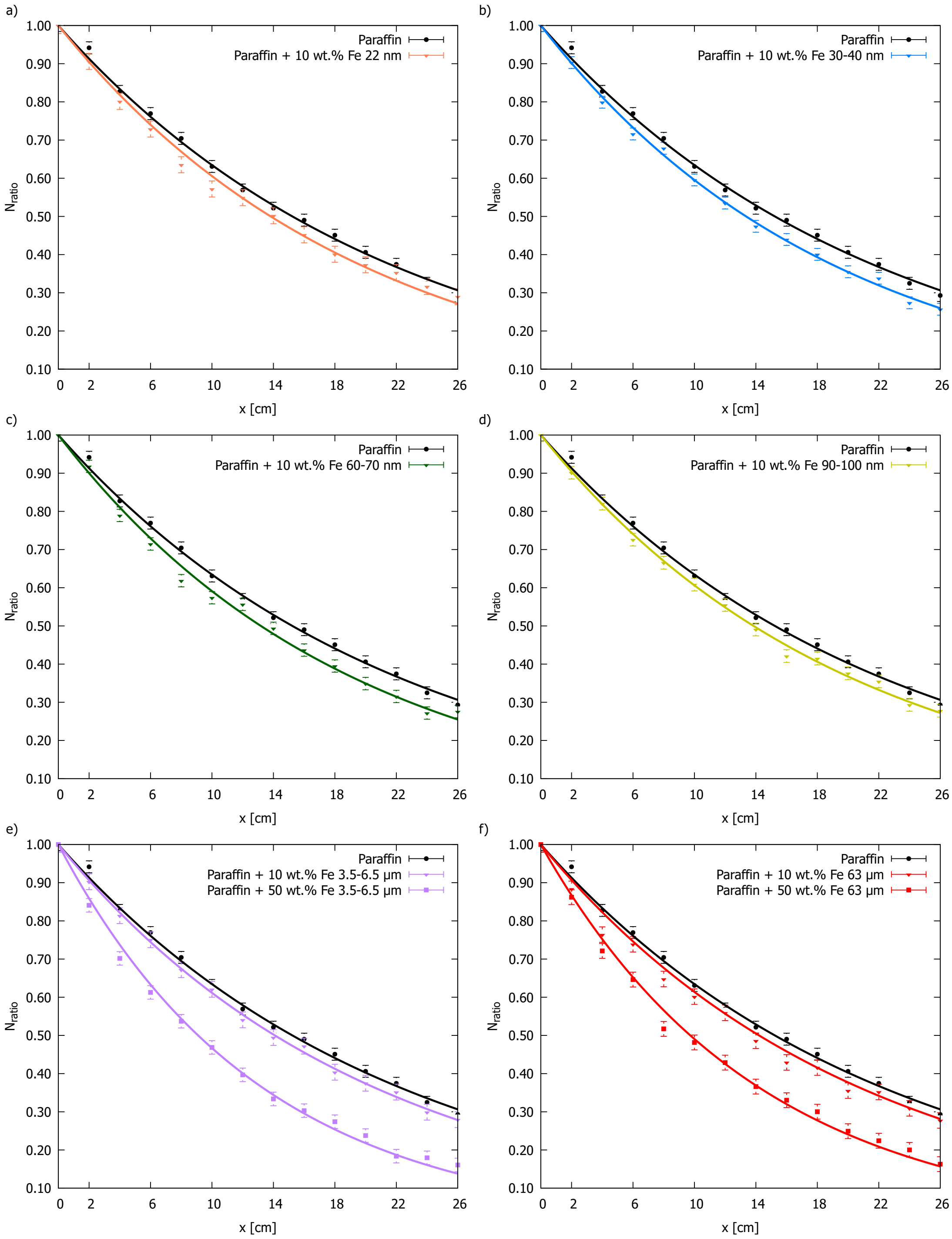

Figure S11: The dependence of the ratio of the number of detected photons for each examined layer thickness to the number of photons collected without any layer on the thickness of the composite for a) Paraffin + 10 wt.% Fe 22 nm, b) Paraffin + 10 wt.% Fe 30-40 nm, c) Paraffin + 10 wt.% Fe 60-70 nm, d) Paraffin + 10 wt.% Fe 90-100 nm, e) Paraffin + 10 wt.% and 50 wt.% Fe 3.5-6.5 μm, and f) Paraffin + 10 wt.% and 50 wt.% Fe 63 μm. Symbols present experimental data, lines present Eq. (3).

Table S13: The summary of measurement data for the manufactured micro/nanocomposites with 10 wt. % of iron nano and microparticles presented in the form of the ratio of the number of counts for a given layer thickness (N) to the number of counts coming from the source (N_0) as the average of three measurement series for each sample.

d [m]	Paraffin + 10 wt.% Fe 22 nm	Paraffin + 10 wt.% Fe 30-40 nm	Paraffin + 10 wt.% Fe 60-70 nm	Paraffin + 10 wt.% Fe 90-100 nm	Paraffin + 10 wt.% Fe 3.5-6.5 μm	Paraffin + 10 wt.% Fe 63 μm
0	1	1	1	1	1	1
0.02	0.906	0.903	0.918	0.901	0.902	0.884
0.04	0.801	0.799	0.789	0.820	0.814	0.764
0.06	0.729	0.716	0.715	0.726	0.751	0.739
0.08	0.635	0.678	0.619	0.665	0.672	0.648
0.10	0.572	0.596	0.574	0.608	0.621	0.602
0.12	0.549	0.535	0.556	0.555	0.541	0.559
0.14	0.502	0.474	0.494	0.491	0.495	0.486
0.16	0.452	0.440	0.437	0.421	0.472	0.429
0.18	0.401	0.401	0.395	0.415	0.404	0.416
0.20	0.373	0.355	0.349	0.376	0.375	0.355
0.22	0.353	0.338	0.315	0.355	0.351	0.352
0.24	0.316	0.274	0.272	0.293	0.299	0.309
0.26	0.290	0.257	0.277	0.278	0.280	0.277

Table S14: The summary of measurement data for the pure paraffin and manufactured microcomposites with 50 wt.% of iron microparticles presented in the form of the ratio of the number of counts for a given layer thickness (N) to the number of counts coming from the source (N_0) as the average of three measurement series for each sample.

d [m]	Paraffin	Paraffin + 50 wt.% Fe 3.5-6.5 μm	Paraffin + 50 wt.% Fe 63 μm
0	1	1	1
0.02	0.942	0.841	0.862
0.04	0.827	0.702	0.721
0.06	0.769	0.613	0.646
0.08	0.704	0.537	0.517
0.10	0.631	0.469	0.482
0.12	0.569	0.397	0.429
0.14	0.521	0.334	0.366
0.16	0.490	0.303	0.330
0.18	0.451	0.274	0.300
0.20	0.406	0.238	0.249
0.22	0.375	0.184	0.224
0.24	0.325	0.180	0.200
0.26	0.293	0.161	0.163

Table S15: Summary of HVL (cm) value and linear attenuation coefficient μ (cm^{-1}) of pure iron and composites in the literature data, together with the microcomposites samples experimentally-designated in this study.

Sample	HVL [cm]	μ [cm^{-1}]
Pure iron (1173 keV) [37]	1.45	0.47803
Pure iron (1333 keV) [37]	1.81	0.38295
Fe_3O_4-PVA-18.5% [13]	1.71	0.40534
Ba-Fe-Ni (1173 keV) [9]	3.82	0.18145
Ba-Fe-Ni (1333 keV) [9]	4.15	0.16702
Nano iron slag-marble-25% (1173 keV) [10]	3.89	0.17818
Nano iron slag-marble-25% (1332 keV) [10]	4.33	0.16008
Micro iron slag-marble-25% (1173 keV) [10]	4.48	0.15472
Micro iron slag-marble-25% (1332 keV) [10]	4.98	0.13918
α-Fe_2O_3-HDPE-40% (1773 keV) [12]	5.09	0.13617
α-Fe_2O_3-HDPE-40% (1333 keV) [12]	5.63	0.12311
Paraffin + 50 wt.% Fe 3.5-6.5 μm (this study)	9.12$\pm$ 0.21	0.0760$\pm$0.0012
Paraffin + 50 wt.% Fe 63 μm (this study)	9.74$\pm$ 0.24	0.0712$\pm$0.0012
Paraffin + 10 wt.% Fe 3.5-6.5 μm (this study)	14.09$\pm$ 0.16	0.04918$\pm$0.00039
Paraffin + 10 wt.% Fe 3.5-6.5 μm (this study)	13.68$\pm$ 0.34	0.05064$\pm$0.00088
Pure paraffin (this study)	15.23$\pm$ 0.22	0.04550$\pm$0.00047

Shielding properties - X-ray

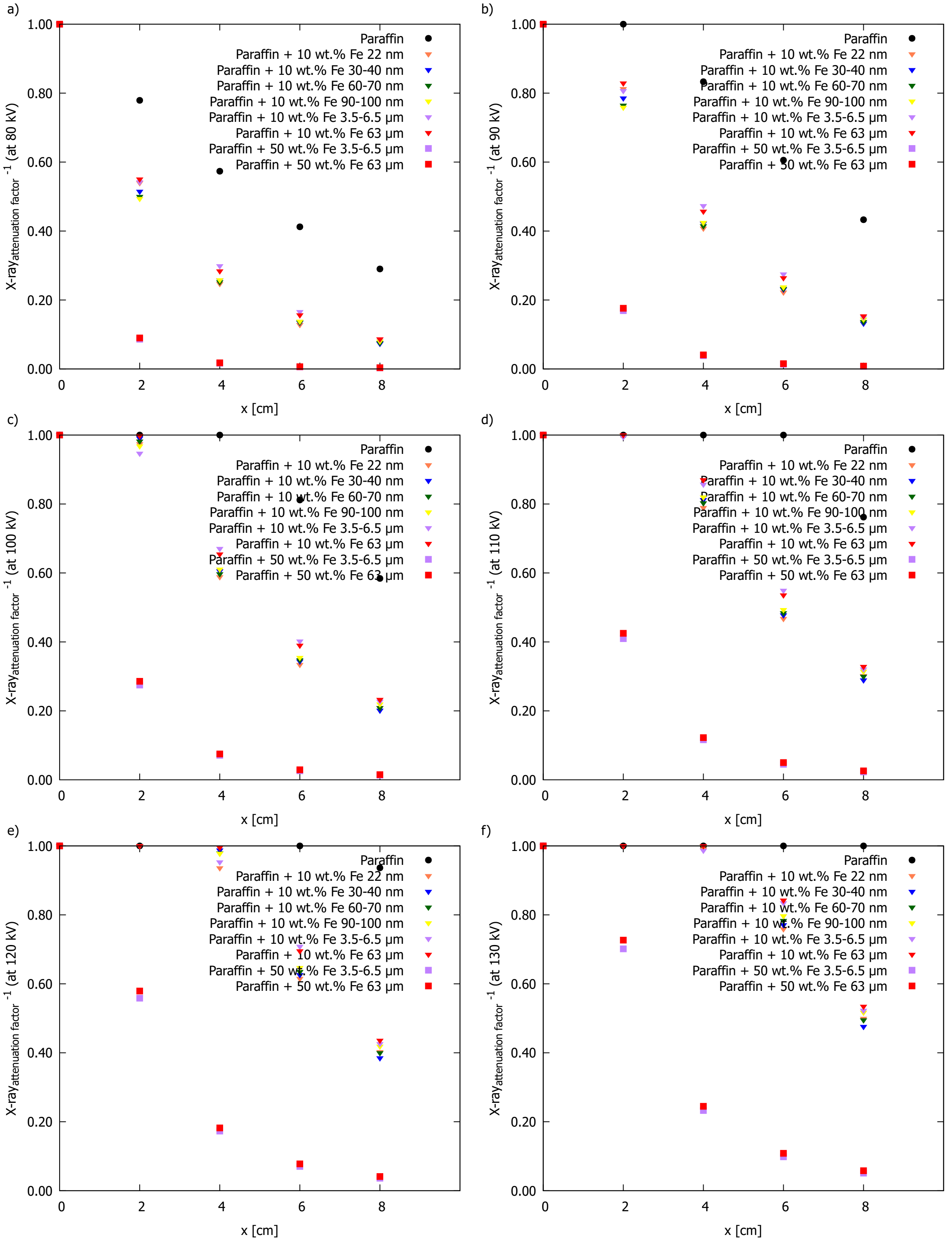

Figure S12: The dependence of X-ray$^{-1}_{attenuation factor}$ for the manufactured nano- and micro-composites with 10% and 50% of iron particles and pure paraffin at 80, 90, 100, 110, 120 and 130 kV voltage.

S7.a Movie

The video "easy formability of shielding nano- and microcomposites" showing the possibility of forming any shape to the manufactured shileding material with the warmth of the hand can be found on the website of the journal.

Table S16: The summary of X-ray shielding measurement data for the manufactured nano- and microcomposites with 10 wt.% of iron particles presented in the form of the ratio of the singal intensity of given material layer thickness (I) to the signal intensity for aired field (I_0) at 70 kV voltage.

d [m]	Paraffin + 10 wt.% Fe 22 nm	Paraffin + 10 wt.% Fe 30-40 nm	Paraffin + 10 wt.% Fe 60-70 nm	Paraffin + 10 wt.% Fe 90-100 nm	Paraffin + 10 wt.% Fe 3.5-6.5 μm	Paraffin + 10 wt.% Fe 63 μm
0	1	1	1	1	1	1
0.02	0.436	0.409	0.398	0.390	0.458	0.459
0.04	0.178	0.186	0.183	0.186	0.238	0.219
0.06	0.085	0.090	0.092	0.093	0.124	0.113
0.08	0.048	0.046	0.048	0.051	0.059	0.058

Table S17: The summary of X-ray shielding measurement data for the manufactured microcomposites with 50 wt.% of iron microparticles and pure paraffin presented in the form of the ratio of the singal intensity of given material layer thickness (I) to the signal intensity for aired field (I_0) at 70 kV voltage.

d [m]	Paraffin	Paraffin + 50 wt.% Fe 3.5-6.5 μm	Paraffin + 50 wt.% Fe 63 μm
0	1	1	1
0.02	0.723	0.049	0.051
0.04	0.514	0.007	0.008
0.06	0.360	0.003	0.003
0.08	0.249	0.002	0.002

Table S18: The summary of X-ray shielding measurement data for the manufactured nano- and microcomposites with 10 wt.% of iron particles presented in the form of the ratio of the singal intensity of given material layer thickness (I) to the signal intensity for aired field (I_0) at 80 kV voltage.

d [m]	Paraffin + 10 wt.% Fe 22 nm	Paraffin + 10 wt.% Fe 30-40 nm	Paraffin + 10 wt.% Fe 60-70 nm	Paraffin + 10 wt.% Fe 90-100 nm	Paraffin + 10 wt.% Fe 3.5-6.5 μm	Paraffin + 10 wt.% Fe 63 μm
0	1	1	1	1	1	1
0.02	0.540	0.515	0.500	0.494	0.542	0.550
0.04	0.247	0.257	0.252	0.258	0.298	0.283
0.06	0.128	0.135	0.136	0.138	0.165	0.156
0.08	0.076	0.074	0.076	0.080	0.085	0.086

Table S19: The summary of X-ray shielding measurement data for the manufactured micro-composites with 50 wt.% of iron microparticles and pure paraffin presented in the form of the ratio of the singal intensity of given material layer thickness (I) to the signal intensity for aired field (I_0) at 80 kV voltage.

d [m]	Paraffin	Paraffin + 50 wt.% Fe 3.5-6.5 μm	Paraffin + 50 wt.% Fe 63 μm
0	1	1	1
0.02	0.791	0.086	0.089
0.04	0.571	0.016	0.017
0.06	0.407	0.005	0.006
0.08	0.288	0.003	0.003

Table S20: The summary of X-ray shielding measurement data for the manufactured nano- and microcomposites with 10 wt.% of iron particles presented in the form of the ratio of the singal intensity of given material layer thickness (I) to the signal intensity for aired field (I_0) at 90 kV voltage.

d [m]	Paraffin + 10 wt.% Fe 22 nm	Paraffin + 10 wt.% Fe 30-40 nm	Paraffin + 10 wt.% Fe 60-70 nm	Paraffin + 10 wt.% Fe 90-100 nm	Paraffin + 10 wt.% Fe 3.5-6.5 μm	Paraffin + 10 wt.% Fe 63 μm
0	1	1	1	1	1	1
0.02	0.812	0.785	0.764	0.758	0.807	0.829
0.04	0.408	0.422	0.415	0.424	0.473	0.457
0.06	0.222	0.231	0.233	0.237	0.275	0.264
0.08	0.137	0.132	0.137	0.144	0.150	0.153

Table S21: The summary of X-ray shielding measurement data for the manufactured micro-composites with 50 wt.% of iron microparticles and pure paraffin presented in the form of the ratio of the singal intensity of given material layer thickness (I) to the signal intensity for aired field (I_0) at 90 kV voltage.

d [m]	Paraffin	Paraffin + 50 wt.% Fe 3.5-6.5 μm	Paraffin + 50 wt.% Fe 63 μm
0	1	1	1
0.02	0.999	0.169	0.176
0.04	0.824	0.038	0.041
0.06	0.598	0.014	0.015
0.08	0.430	0.007	0.008

Table S22: The summary of X-ray shielding measurement data for the manufactured nano- and microcomposites with 10 wt.% of iron particles presented in the form of the ratio of the singal intensity of given material layer thickness (I) to the signal intensity for aired field (I_0) at 100 kV voltage.

d [m]	Paraffin + 10 wt.% Fe 22 nm	Paraffin + 10 wt.% Fe 30-40 nm	Paraffin + 10 wt.% Fe 60-70 nm	Paraffin + 10 wt.% Fe 90-100 nm	Paraffin + 10 wt.% Fe 3.5-6.5 μm	Paraffin + 10 wt.% Fe 63 μm
0	1	1	1	1	1	1
0.02	0.974	0.991	0.982	0.968	0.946	0.997
0.04	0.588	0.606	0.597	0.611	0.670	0.654
0.06	0.334	0.344	0.348	0.354	0.402	0.389
0.08	0.209	0.201	0.209	0.219	0.227	0.232

Table S23: The summary of X-ray shielding measurement data for the manufactured micro-composites with 50 wt.% of iron microparticles and pure paraffin presented in the form of the ratio of the singal intensity of given material layer thickness (I) to the signal intensity for aired field (I_0) at 100 kV voltage.

d [m]	Paraffin	Paraffin + 50 wt.% Fe 3.5-6.5 μm	Paraffin + 50 wt.% Fe 63 μm
0	1	1	1
0.02	0.999	0.274	0.285
0.04	0.999	0.071	0.075
0.06	0.802	0.026	0.029
0.08	0.583	0.013	0.015

Table S24: The summary of X-ray shielding measurement data for the manufactured nano- and microcomposites with 10 wt.% of iron particles presented in the form of the ratio of the singal intensity of given material layer thickness (I) to the signal intensity for aired field (I_0) at 110 kV voltage.

d [m]	Paraffin + 10 wt.% Fe 22 nm	Paraffin + 10 wt.% Fe 30-40 nm	Paraffin + 10 wt.% Fe 60-70 nm	Paraffin + 10 wt.% Fe 90-100 nm	Paraffin + 10 wt.% Fe 3.5-6.5 μm	Paraffin + 10 wt.% Fe 63 μm
0	1	1	1	1	1	1
0.02	0.999	0.999	0.999	0.999	0.994	0.999
0.04	0.789	0.812	0.803	0.820	0.857	0.870
0.06	0.467	0.478	0.484	0.493	0.549	0.536
0.08	0.302	0.289	0.300	0.315	0.321	0.328

Table S25: The summary of X-ray shielding measurement data for the manufactured microcomposites with 50 wt.% of iron microparticles and pure paraffin presented in the form of the ratio of the singal intensity of given material layer thickness (I) to the signal intensity for aired field (I_0) at 110 kV voltage.

d [m]	Paraffin	Paraffin + 50 wt.% Fe 3.5-6.5 μm	Paraffin + 50 wt.% Fe 63 μm
0	1	1	1
0.02	0.999	0.409	0.425
0.04	0.999	0.116	0.122
0.06	0.999	0.045	0.050
0.08	0.755	0.023	0.026

Table S26: The summary of X-ray shielding measurement data for the manufactured nano- and microcomposites with 10 wt.% of iron particles presented in the form of the ratio of the singal intensity of given material layer thickness (I) to the signal intensity for aired field (I_0) at 120 kV voltage.

d [m]	Paraffin + 10 wt.% Fe 22 nm	Paraffin + 10 wt.% Fe 30-40 nm	Paraffin + 10 wt.% Fe 60-70 nm	Paraffin + 10 wt.% Fe 90-100 nm	Paraffin + 10 wt.% Fe 3.5-6.5 μm	Paraffin + 10 wt.% Fe 63 μm
0	1	1	1	1	1	1
0.02	0.999	0.999	0.999	0.999	0.999	0.999
0.04	0.936	0.988	0.979	0.978	0.953	0.995
0.06	0.615	0.626	0.635	0.647	0.708	0.695
0.08	0.403	0.385	0.400	0.418	0.426	0.436

Table S27: The summary of X-ray shielding measurement data for the manufactured micro-composites with 50 wt.% of iron microparticles and pure paraffin presented in the form of the ratio of the singal intensity of given material layer thickness (I) to the signal intensity for aired field (I_0) at 120 kV voltage.

d [m]	Paraffin	Paraffin + 50 wt.% Fe 3.5-6.5 μm	Paraffin + 50 wt.% Fe 63 μm
0	1	1	1
0.02	0.999	0.558	0.579
0.04	0.999	0.173	0.182
0.06	0.999	0.070	0.078
0.08	0.930	0.036	0.041

Table S28: The summary of X-ray shielding measurement data for the manufactured nano- and microcomposites with 10 wt.% of iron particles presented in the form of the ratio of the singal intensity of given material layer thickness (I) to the signal intensity for aired field (I_0) at 130 kV voltage.

d [m]	Paraffin + 10 wt.% Fe 22 nm	Paraffin + 10 wt.% Fe 30-40 nm	Paraffin + 10 wt.% Fe 60-70 nm	Paraffin + 10 wt.% Fe 90-100 nm	Paraffin + 10 wt.% Fe 3.5-6.5 μm	Paraffin + 10 wt.% Fe 63 μm
0	1	1	1	1	1	1
0.02	0.999	0.999	0.999	0.999	0.999	0.999
0.04	0.990	0.999	0.999	0.999	0.987	0.999
0.06	0.759	0.771	0.783	0.797	0.834	0.842
0.08	0.499	0.476	0.495	0.517	0.523	0.534

Table S29: The summary of X-ray shielding measurement data for the manufactured micro-composites with 50 wt.% of iron microparticles and pure paraffin presented in the form of the ratio of the singal intensity of given material layer thickness (I) to the signal intensity for aired field (I_0) at 130 kV voltage.

d [m]	Paraffin	Paraffin + 50 wt.% Fe 3.5-6.5 μm	Paraffin + 50 wt.% Fe 63 μm
0	1	1	1
0.02	0.999	0.701	0.727
0.04	0.999	0.233	0.245
0.06	0.999	0.098	0.108
0.08	0.999	0.051	0.058
